# CD41-deficient exosomes from non-traumatic femoral head necrosis tissues impair osteogenic differentiation and migration of mesenchymal stem cells

**DOI:** 10.1038/s41419-020-2496-y

**Published:** 2020-04-27

**Authors:** Weiwen Zhu, MinKang Guo, Wu Yang, Min Tang, Tingmei Chen, Delu Gan, Dian Zhang, Xiaojuan Ding, Anping Zhao, Pei Zhao, Wenlong Yan, Jian Zhang

**Affiliations:** 1 Department of Orthopedics, The First Affiliated Hospital of Chongqing Medical University, Chongqing, 400016 China; 2 Key Laboratory of Diagnostic Medicine Designated by the Ministry of Education, Chongqing Medical University, Chongqing, 400016 China; 3 Department of Laboratory, The Second Affiliated Hospital of Chongqing Medical University, Chongqing, 400016 China

**Keywords:** Stem-cell differentiation, Stem-cell differentiation, Diseases

## Abstract

Non-traumatic osteonecrosis of the femoral head (ONFH) is clinically a devastating and progressive disease without an effective treatment. Mesenchymal stem cells (MSCs) transplantation has been used to treat ONFH in early stage, but the failure rate of this therapy is high due to the reduced osteogenic differentiation and migration of the transplanted MSCs related with pathological bone tissues. However, the mechanism responsible for this decrease is still unclear. Therefore, we assume that the implanted MSCs might be influenced by signals delivered from pathological bone tissue, where the exosomes might play a critical role in this delivery. This study showed that exosomes from ONFH bone tissues (ONFH-exos) were able to induce GC-induced ONFH-like damage, in vivo and impair osteogenic differentiation and migration of MSCs, in vitro. Then, we analyzed the differentially expressed proteins (DEPs) in ONFH-exos using proteomic technology and identified 842 differentially expressed proteins (DEPs). On the basis of gene ontology (GO) enrichment analysis of DEPs, fold-changes and previous report, cell adhesion-related CD41 (integrin α2b) was selected for further investigation. Our study showed that the CD41 (integrin α2b) was distinctly decreased in ONFH-exos, compared to NOR-exos, and downregulation of CD41 could impair osteogenic differentiation and migration of the MSCs, where CD41-integrin β3-FAK-Akt-Runx2 pathway was involved. Finally, our study further suggested that CD41-affluent NOR-exos could restore the glucocorticoid-induced decline of osteogenic differentiation and migration in MSCs, and prevent GC-induced ONFH-like damage in rat models. Taken together, our study results revealed that in the progress of ONFH, exosomes from the pathological bone brought about the failure of MSCs repairing the necrotic bone for lack of some critical proteins, like integrin CD41, and prompted the progression of experimentally induced ONFH-like status in the rat. CD41 could be considered as the target of early diagnosis and therapy in ONFH.

## Introduction

Non-traumatic osteonecrosis of the femoral head (ONFH) is a common disease characterized by rapid procession, high disability rate and severe influence on life quality^1,2^. In the US, 10,000–20,000 patients are newly found to be affected with the disease per year^3^ and in China, 100,000–200,000 new cases were reported per year^4^. Various medical treatments have been introduced, but no pharmacological prevention or treatment of osteonecrosis is recommendable^5^. Mesenchymal stem cells (MSCs) transplantation has been used to treat the ONFH in early stage, but the failure rate is about 30%. Most of the patients have to undergo a total hip arthroplasty (THA)^6^. Therefore, ONFH affects the patients physically and psychologically and brings a huge financial burden on family and society. The difficulties in diagnosis and treatment for this disease mainly result from the unclear underlying mechanism.

MSCs with pluripotency of differentiation play a critical role in ONFH^7,8^. The impaired osteogenic differentiation and migration of MSCs is considered as the major factor leading to ONFH^9^. However, MSCs implantation remains a high rate of failure^10^. On one hand, the osteogenic differentiation and migration of implanted MSCs is decreased, which seem to be assimilated by the pathological tissue. That might be one of the main causes for the failure of cytotherapy. On the other hand, it is noticeable that the progress of ONFH is unlikely to be halted by blocking the exposure to the risk factors. Therefore, we suppose that ONFH bone tissue might deliver some signals to affect the normal tissues and drive the disease progress via an undetected approach. Exploring the delivery approach in ONFH is important to find out the target for early diagnosis and treatment.

As we know, intercellular signaling delivery is mainly mediated by soluble cytokines, chemokines, and other soluble factors; however, extracellular vesicles (EV) have become recognized as new mediators in this process^11^. Exosome is a member of EV, with the size of 40–150 nm. It packages the proteins and genetic materials from host cells into lipid bilayer and delivers this cargo to the recipient cells^12,13^. This communicational approach can increase the spreading scope and duration time of the signals. Recently, these nanoparticles were reported to induce some diseases, like chronic obstructive pulmonary disease and coronary heart disease^14,15^. However, the role of exosomes in ONFH is still confusing and the feasibility of exosomes in treating this disease remains unclear. Due to all the cells in tissues surrounded by exosomes, cells co-cultured with exosomes from the bone tissue of ONFH (ONFH-exos) approximate those in original environment. Whereas, it is difficult to obtain the bone tissues from ONFH patients, study on ONFH-exos has never been reported before. Thus, it is necessary to study the effect of ONFH-exos.

Our study disclosed that the injection of ONFH-exos could induce incidence of GC-induced ONFH-like damage in vivo and suppress the osteogenic differentiation and migration of MSCs. To explore the change of osteogenic and migratory factors, we analyzed the proteome of ONFH-exos and demonstrated the importance of CD41 (integrin α2b) in osteogenic differentiation and migration of MSCs. Furthermore, we showed the reduction of CD41 in ONFH-exos and found out the potential molecular mechanism in ONFH induced by exosomes. These results demonstrated that ONFH tissue facilitated the disease progress through spreading the signaling included in exosomes to impair the osteoblast differentiation and migration of MSCs, and that CD41 played an important role in this process.

## Results

### Demographic characteristics of patients and characteristics of exosomes from all samples

**Fig. 1:**
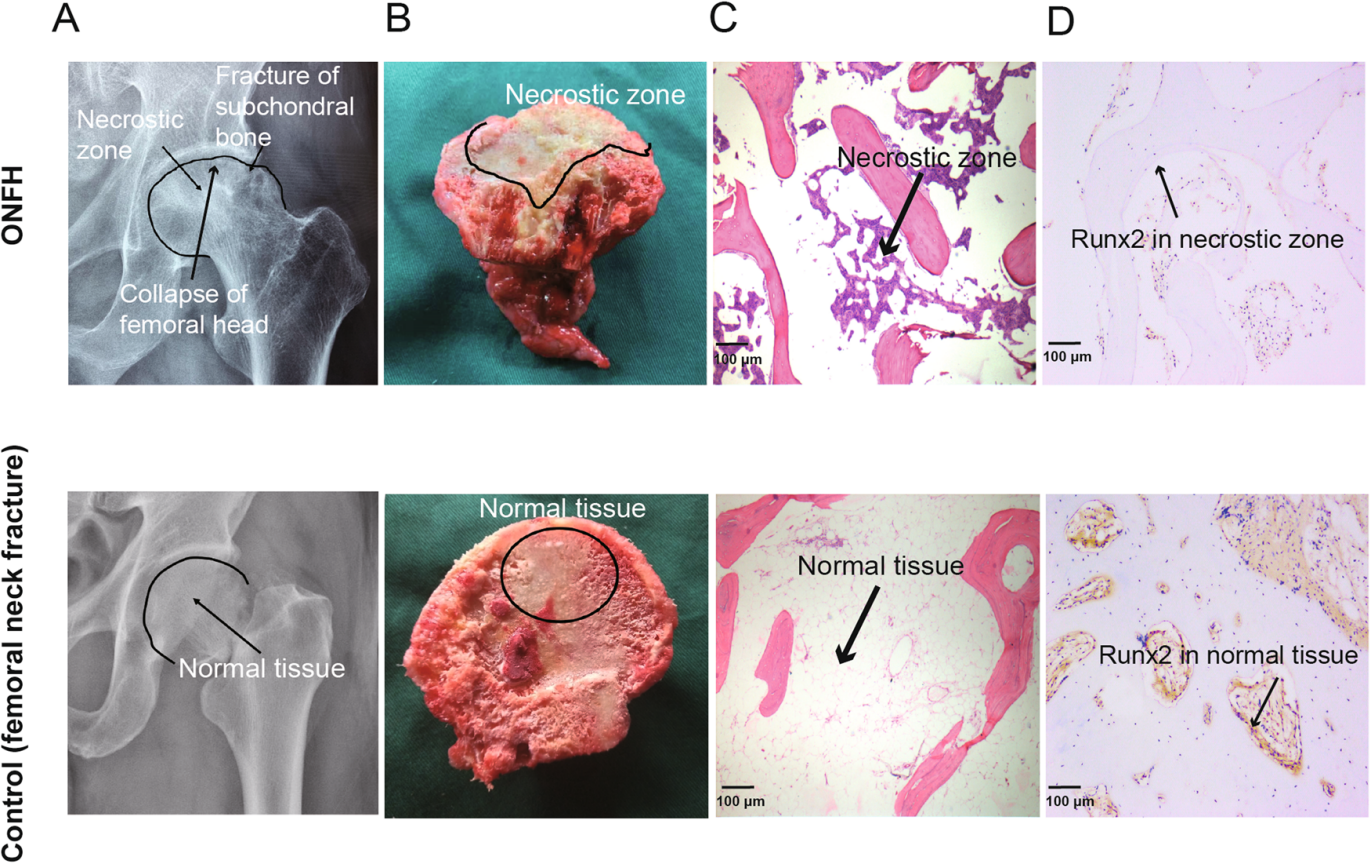
Radiographs, gross specimens and histological images of samples. The radiographs, gross specimen and histological image of an ONFH patient and a patient with femoral neck fracture (control) was shown above. **a** The X-ray image of ONFH patient exhibited the fracture of subchondral bone, necrotic zone and collapse of femoral head, compared to the control. **b** The gross specimen of ONFH sample showed fracture of subchondral bone, homogeneous change in the focal necrotic zone, increase of fat, change of structure and collapse of femoral head, in femoral head. **c** Histological image of ONFH sample displayed abnormal cancellous bones in ONFH sample and homogenous necrotic material. **d** IHC staining of ONFH sample showed a remarkable reduction of Runx2 in ONFH sample.

The demographics of recruited subjects are shown in Table S1. No significant difference was seen in gender, age and BMI between ONFH and control patients. According to the Association Research Circulation Osseuse (ARCO) classification system^16^, all the selected ONFH patients were classified into stage III or IV. To confirm the diagnosis, the radiography, gross specimen and histopathology image were obtained from each subject. The typical characteristics of an ONFH sample were exhibited in Fig. 1. In Fig. 1a, the fracture of subchondral bone, necrosis zone and collapse of femoral head are presented in an ONFH radiograph. In Fig. 1b, in addition to the pathological changes indicated in radiograph, increase of fat and change of structure in femoral head are presented in gross specimen. Histological image displays abnormal cancellous bone and the homogenous necrotic material in bone marrow (Fig. 1c). Consistent with previous report^17^, the expression of Runt-related transcription factor 2 (Runx2), a marker of osteogenic differentiation, is notably declined in ONFH bone tissue (Fig. 1d). All of these data conformed to the diagnosis of ONFH by ARCO classification, and IHC slice showed the decline of osteogenic differentiation.

**Fig. 2:**
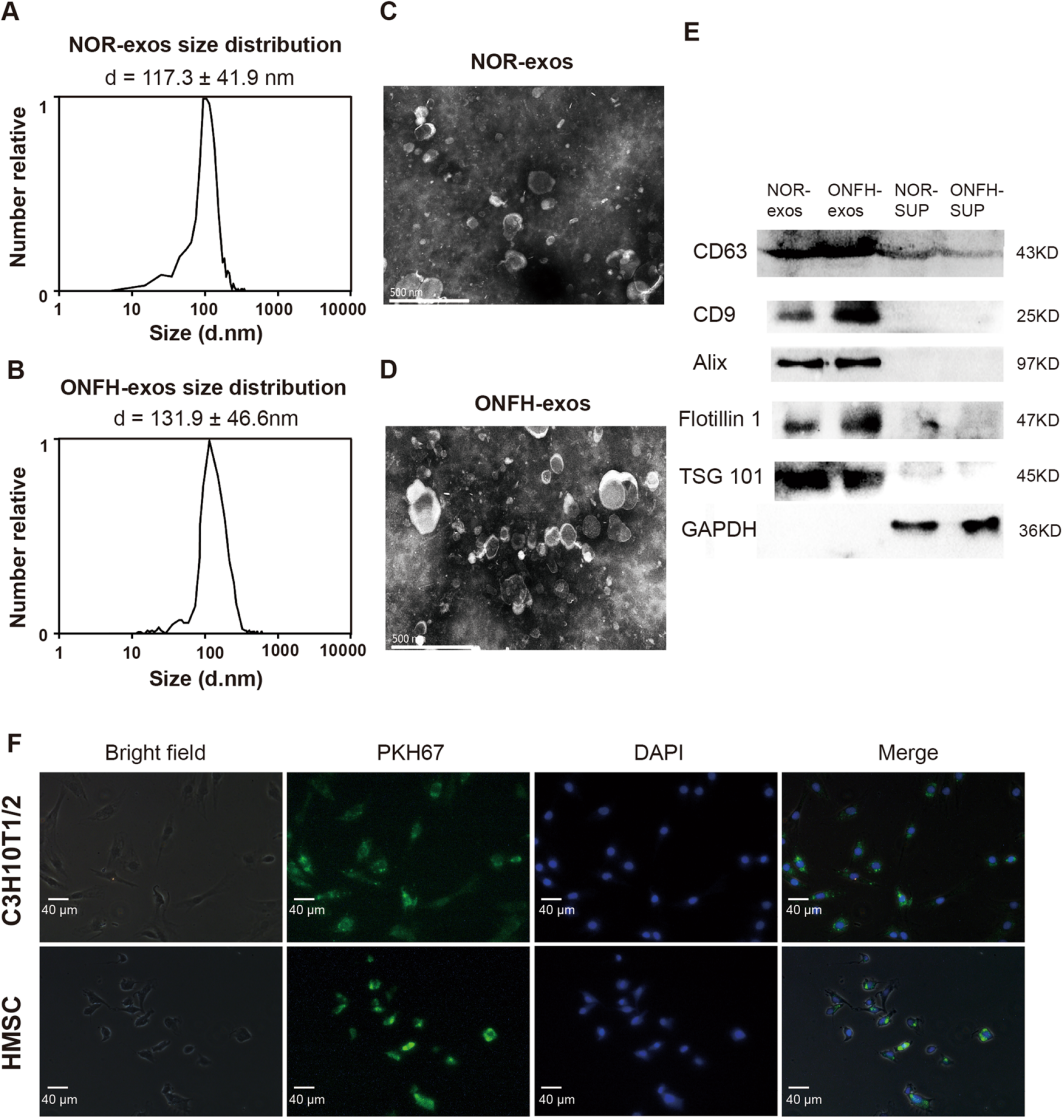
Characterization of ONFH-exos and NOR-exos and uptake assay of exosomes. **a**, **b** Particle size distribution measured by NTA showed that the diameter distribution range of NOR-exos was 117 ± 41.9 nm and diameter distribution range of ONFH-exos was 131.9 ± 46.6 nm. “d”: diameter. **c**, **d** Transmission electron microscopy images displayed the double membrane and discoid shape of NOR-exos and ONFH-exos. Scale bar: 500 nm. **e** Western blotting analysis revealed that exosomal markers, CD63, CD9, Alix, Flotillin-1 and TSG101 were incremental in NOR-exos and ONFH-exos, compared to the supernatants. The normal bone tissue supernatant (NOR-SUP) and ONFH bone tissues supernatant (ONFH-SUP) in second ultra-centrifugation were used as control. **f** The uptake test showed that the exosomes were taken in by C3H10T1/2 cells and HMSCs, at 12 h and 6 h, respectively. PKH67 was used to stain the exosomes, and DAPI was used to stain the nuclei.

To characterize the purified exosome fractions, NTA, TEM and the exosome markers analysis were conducted. The results from NTA showed diameter distribution with an average dimension of 117.3 ± 41.9 nm in NOR-exos and 131.9 ± 46.6 nm in ONFH-exos (Fig. 2a, b), which was consistent with previous study^18^. The yield of exosomes in ONFH tissues is 5.38 ± 1.75 (10^11^/g) and the yield of exosomes in normal tissues is 2.8 ± 0.85 (10^11^/g), which indicated the ONFH tissues generated more exosomes (Table S1). TEM imaging displayed that the exosomes with round shape in morphology and double-membrane structure, had diameters ranging from 40 to 150 nm (Fig. 2c, d). Moreover, western blotting results exhibited the exosomal markers—CD63, CD9, Alix, Flotillin1, and TSG101—were abundant in the pellets (Fig. 2e). As the nuclei and mitochondria are the common contaminants in the exosomes isolated by ultracentrifugation, we measured the lamin A and mitofilin and found that these two proteins were lower expressed in the NOR-exos and ONFH-exos (Fig. S1). To test whether the exosomes can be internalized by the host cells, we conducted an uptake assay, and the results showed the exosomes were internalized by C3H10T1/2 cells for 12 h and internalized by HMSCs for 6 h (Fig. 2f). These data suggested that exosomes were successfully separated from the bone samples and could be taken in by C3H10T1/2 cells and HMSCs.

### ONFH-exos cause the incidence of GC-induced ONFH-like damage in vivo

**Fig. 3:**
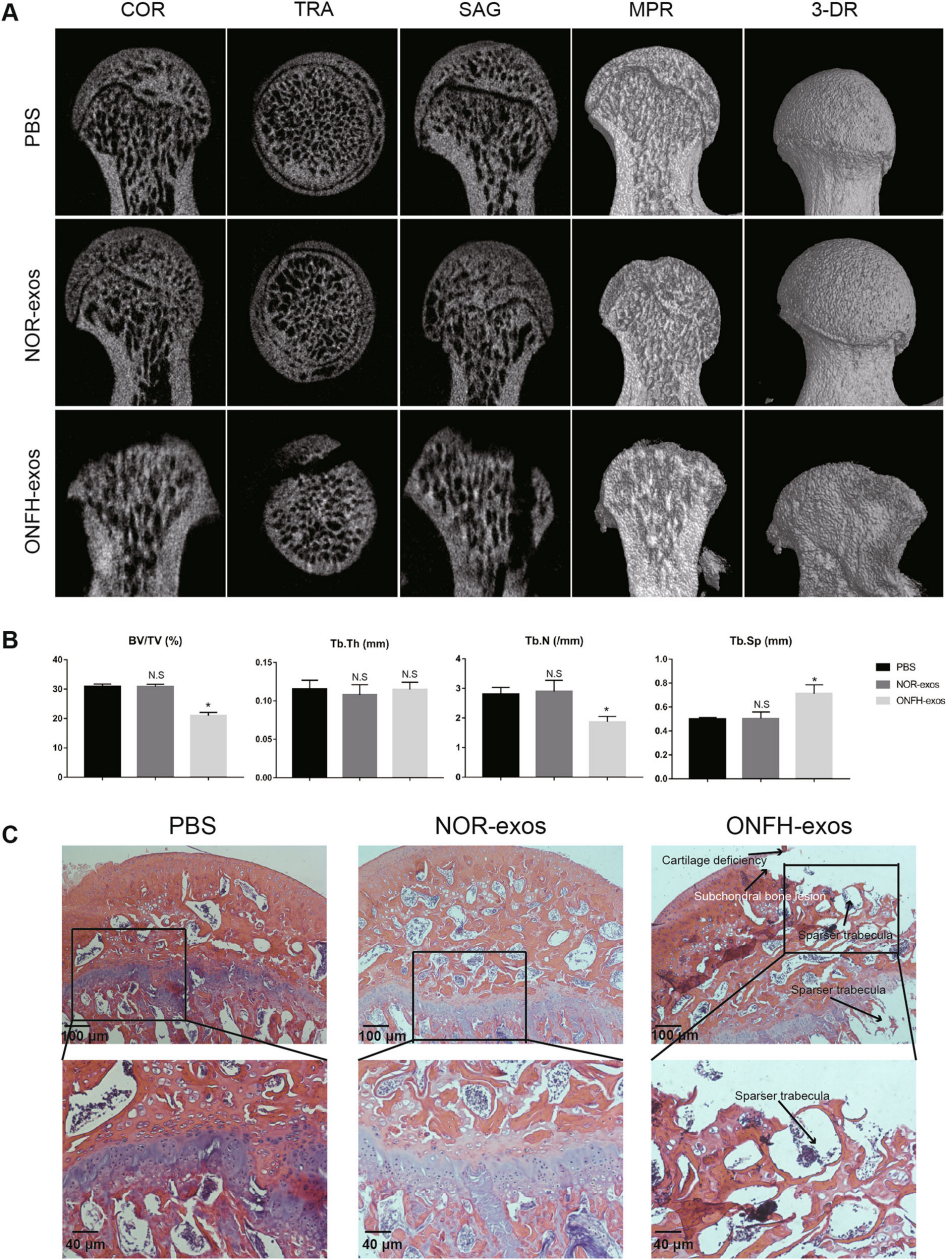
The effect of ONFH-exos in S-D rats. **a** COR, TRA, SAG, MPR, and 3-DR images of rat femoral heads were obtained using Micro-CT scanning. In ONFH-exos group, the Micro-CT scanning image showed cartilage deficiency, subchondral bone lesion and malformation of femoral head, compared to NOR-exos group. **b** Quantitative analysis of osteogenic parameters presented that BV/TV and Tb.N were decreased, Tb.Sp was increased and Tb.Th was unchanged in ONFH-exos group compared to NOR-exos. c HE staining of the femoral heads displayed that ONFH-exos could induce ONFH in rats. The images of ONFH-exos group showed the loss of cartilage deficiency, subchondral bone lesion and sparser trabecular. *P < 0.05, versus PBS group; N.S, no significance versus PBS group. BV/TV, bone volume per tissue volume; Tb.N, trabecular number; Tb.Sp, trabecular separation; Tb.Th, Trabecular thickness. COR, coronal; TRA, transverse; SAG, sagittal; MPR, multiplanar reconstruction; 3-DR, three-dimensional reconstruction. All data were expressed as mean ± SEM.

Next, we injected ONFH-exos, NOR-exos and PBS into 10 rats via tail vein, respectively, to explore the effects caused by ONFH-exos, in vivo. Two months after treatment, the micro-CT scanning results displayed that about 50% rats in ONFH-exos group had bone tissue changes, including cartilage deficiency, subchondral bone lesion and malformed shape, compared to the rats in NOR-exos (Fig. 3a). Qualitative analyses of all the micro-CT parameters showed that BV/TV and Tb.N were decreased, Tb.Sp was increased, but Tb.Th was unvaried in ONFH-exos group (Fig. 3b), which indicated the reduction of the trabecula volume and thickness, the increase of marrow cavity separation in femoral heads. Consistent with the above findings, HE staining (Fig. 3c) revealed the decrease of osteogenesis in the ONFH-exos group. In ONFH-exos group, about 50% rats had GC-induced ONFH-like damage showed as cartilage deficiency, subchondral bone lesion and sparser trabecula. These data suggested that ONFH-exos induced the GC-induced ONFH-like damage and the decline of osteogenesis in vivo.

### Effect of ONFH-exos on osteoblastic differentiation in vitro

**Fig. 4:**
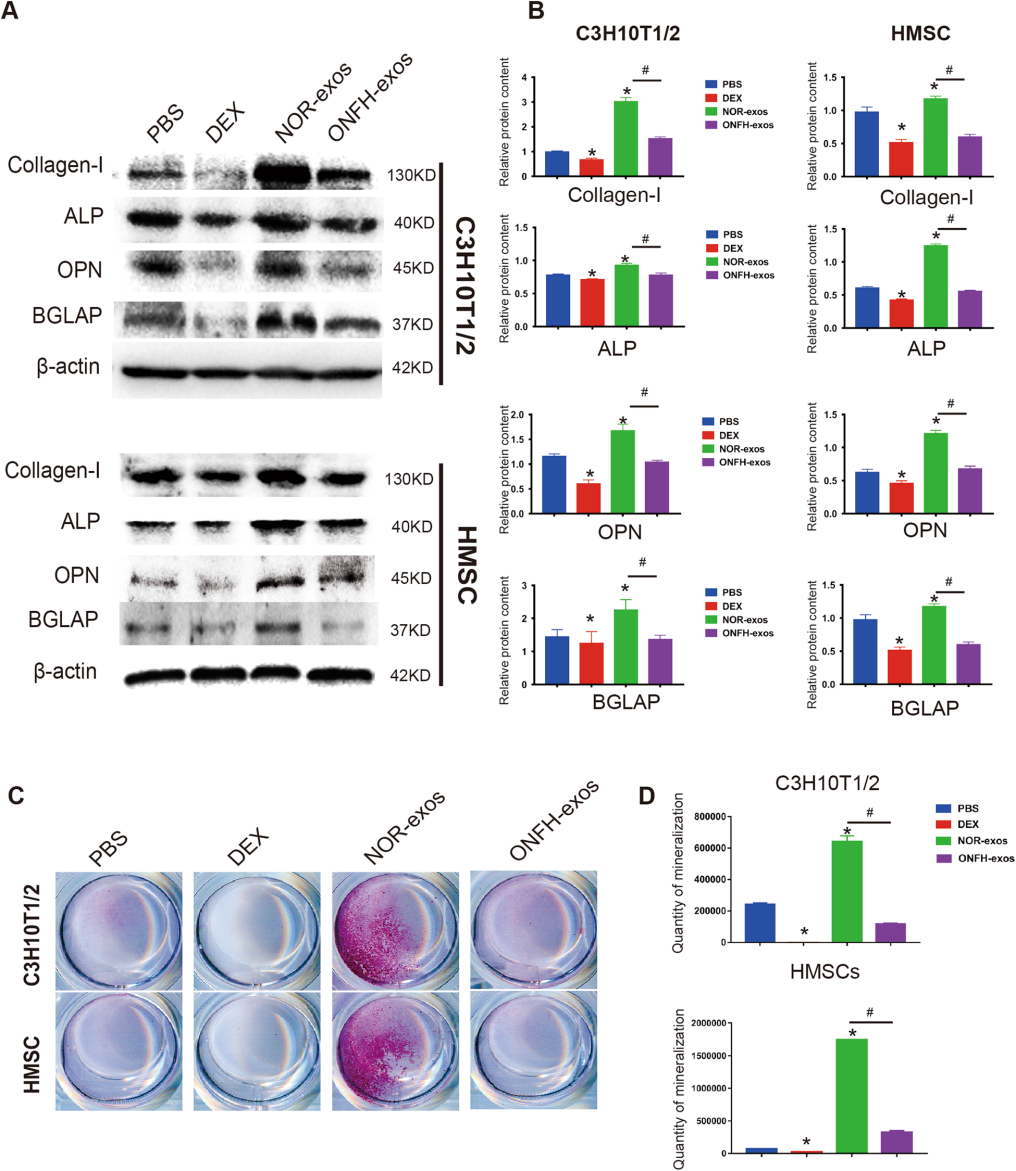
Effect of ONFH-exos on osteogenic differentiation in C3H10T1/2 cells and HMSCs. Osteogenesis of C3H10T1/2 cells and HMSCs was induced by osteogenic differentiation medium. **a** The expression of osteogenic biomarkers, including: ALP, BGLAP, OPN, and COL1 were measured using western blotting analysis. The results showed that the osteogenic biomarkers in ONFH-exos were increased, compared to NOR-exos. **b** Statistical analysis was performed to measure the results in (**a**). **c** Alizarin red S staining presented the formation of calcium mineral deposits. The results showed the remarkable decrease in ONFH-exos, compared to NOR-exos. **d** Statistical analysis was performed to measure the results in (**c**). *P < 0.05, versus PBS group; ^#^*P* < 0.05, versus NOR-exos group. All data were expressed as mean ± SEM.

According to previous studies, ONFH was associated with MSCs, and the osteoblast differentiation of MSCs was declined in this disease^19,20^. Consequently, we assumed that ONFH-exos might drive the ONFH progress through influencing the osteogenic differentiation. To validate our hypothesis, we examined the osteogenic markers, including ALP, BGLAP, OPN and COL1, in MSCs pretreated with PBS, DEX, NOR-exos and ONFH-exos for 48 h (Fig. 4). The western blotting results showed that compared with NOR-exos group, ONFH-exos suppressed the expression of ALP, BGLAP, OPN and COL1 in C3H10T1/2 cells and HMSCs (Fig. 4a, b). In addition, alizarin red S staining were performed after 14-day culture in ODM; the results also exhibited the decline of calcium deposit in ONFH-exos group (Fig. 4c, d). Collectively, these data suggested that ONFH-exos could impair osteogenic differentiation of MSCs. Both osteogenesis and adipogenesis are closely correlated to each other in MSCs differentiation, and DEX was reported be an inducer of MSCs adipogenic differentiation^21^. So, we next investigated the effect of ONFH-exos on adipogenic differentiation of C3H10T1/2 cells (Fig. S2), and the results showed that the adipogenesis of MSCs was facilitated in ONFH-exos group.

### Effect of ONFH-exos on migration

**Fig. 5:**
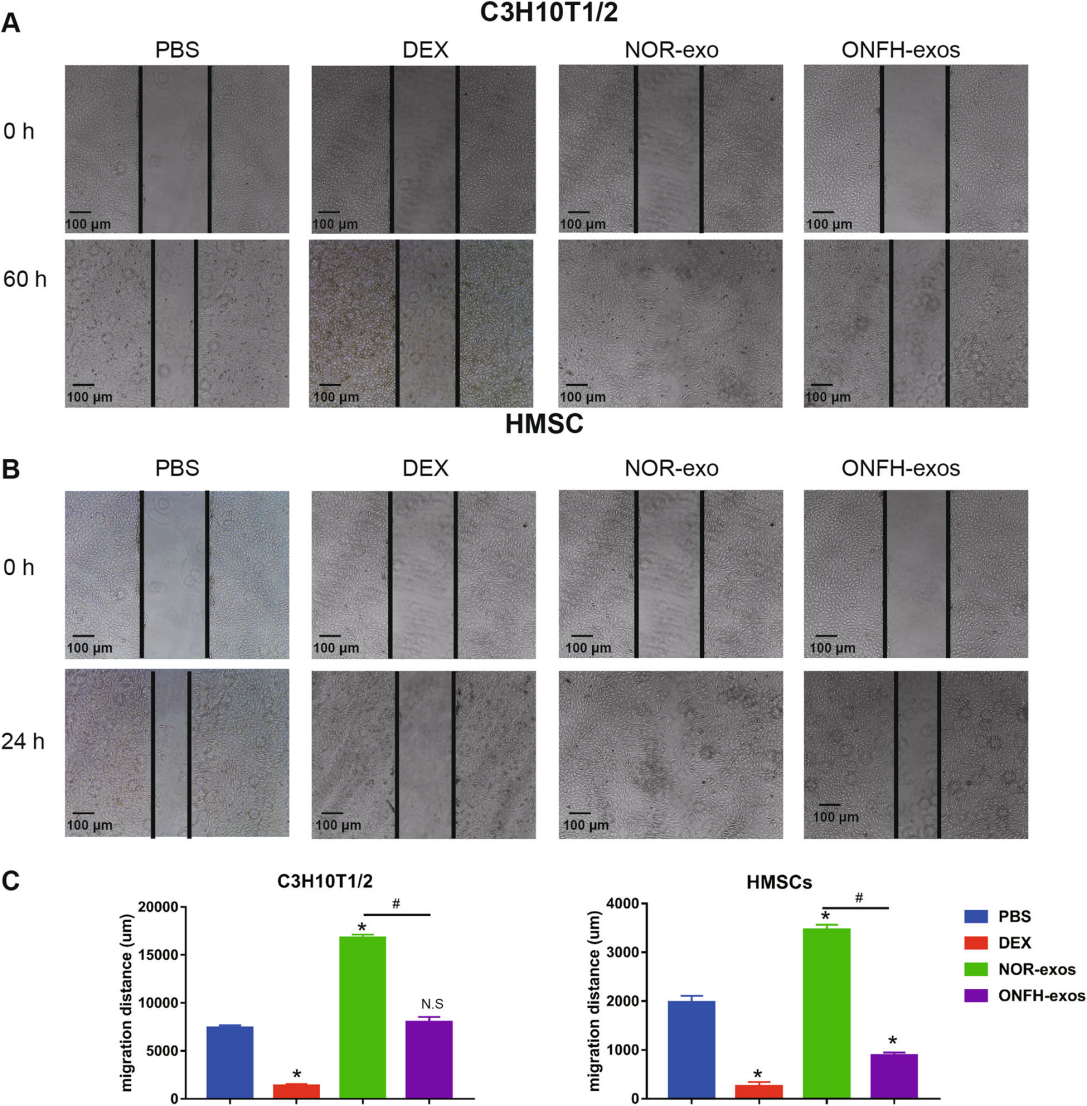
Effect of NOR-exos and ONFH-exos on migration in C3H10T1/2 cells and HMSCs. The migration ability of C3H10T1/2 cells and HMSCs in PBS, DEX, NOR-exos, ONFH-exos was evaluated by wound healing test. **a** The migration of C3H10T1/2 cells was examined at 0 h and 60 hour. **b** The migration of HMSCs were recorded at 0 h and 24 h. **a** and **b** displayed the decline of cell migration in ONFH-exos group, with comparison of NOR-exos. **c** Quantitative analysis was used to evaluate the results of (**a**) and (**b**). *P < 0.05, versus PBS group; ^#^*P* < 0.05, versus NOR-exos group. N.S, no significance. All data were expressed as mean ± SEM.

Most of the MSCs exist in the femoral neck^20^, which migrate to the femoral head and differentiate to osteoblasts for remedying the damaged bone tissue. Consequently, we asked whether ONFH-exos affected MSCs migration. Next, we measured the migration of MSCs in ONFH-exos group employing a wound healing assay and observed the migration of C3H10T1/2 cells and HMSCs every 4 h. The photos reported at 0 h and terminal points (60 h in C3H10T1/2 cells and 24 h in HMSCs) were displayed in Fig. 5a, b. The quantitative data of Fig. 5a, b was displayed in Fig. 5c. The results showed that migratory distance of HMSCs and C3H10T1/2 cells in ONFH-exos group was decreased, compared with NOR-exos. These data indicated that ONFH-exos influenced osteogenic differentiation and migration in MSCs.

The damaged self-repair capacity in ONFH might be associated with the changed number and the feeble viability of MSCs in femoral neck^22^. Therefore, we next examined the effect of ONFH-exos on proliferative activity and apoptosis in C3H10T1/2 cells and HMSCs (Fig. S3). The data illustrated that ONFH-exos were a potent stimuli for the augment of MSCs number by fortifying proliferation and reducing apoptosis.

### Proteomic analysis of ONFH-exos based on iTRAQ

**Fig. 6:**
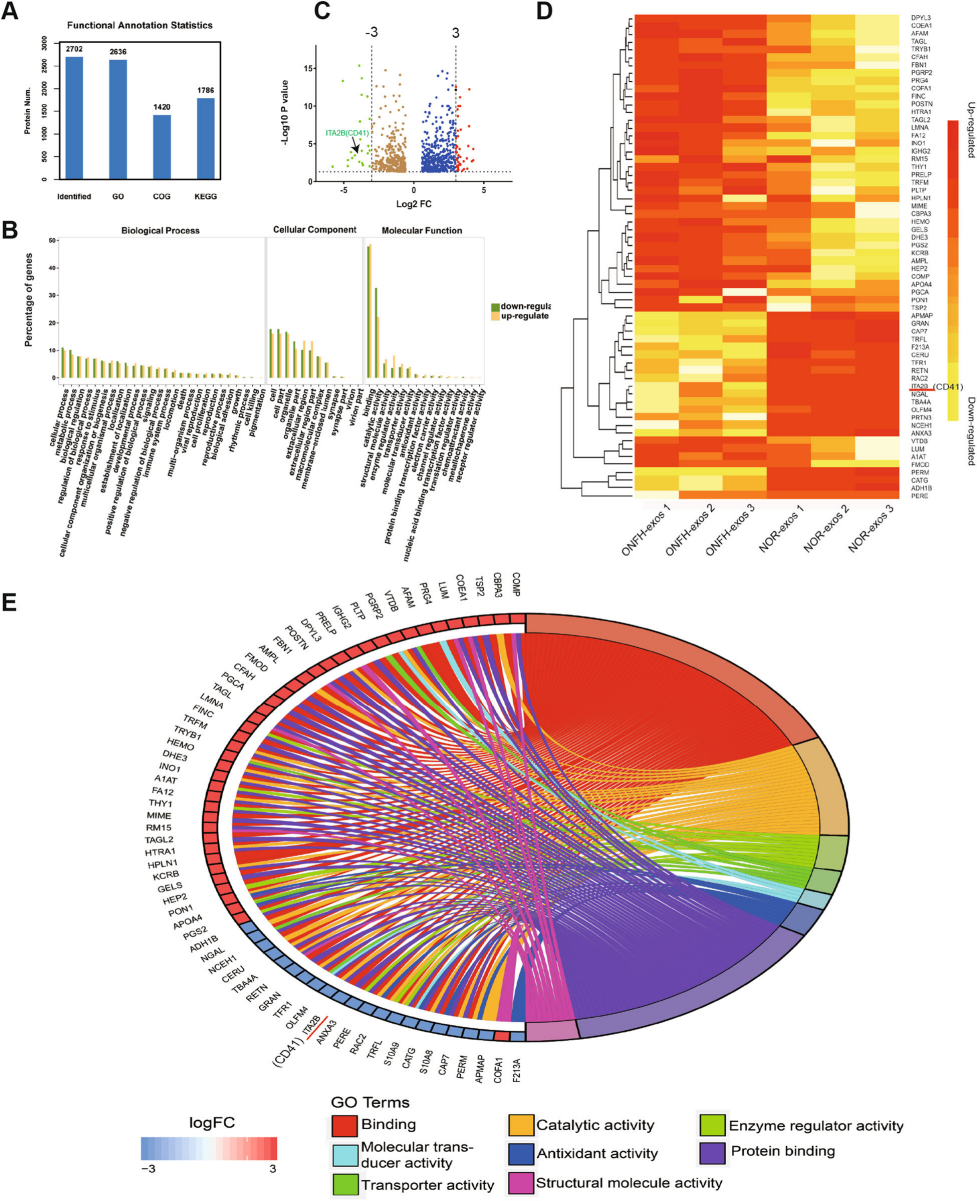
GO analysis of the identified DEPs and cluster analysis of notable DEPs. **a** 2702 proteins were identified, including 2636 in GO, 1420 in COG and 1786 in KEGG. Num: Number. **b** The whole categories of DEPs were enriched in biological process, cellular components, and molecular functions using GO enrichment. In the molecular function analysis, 49.63% of the DEPs were associated with binding. **c** Volcano plot showed all the DEPs, ITA2B (CD41) was included in the notable DEPs with fold decrease of more than 8. FC, fold change. **d** Cluster analysis was performed in notable DEPs, the results were showed in heatmap. **e** Function analysis of notable DEPs was further conducted, the results were presented in a Circos diagram. Most of the notable DEPs were still correlated to binding.

On the basis of Figs. 3–5, it was demonstrated that exosomes from necrotic zone might influence osteoblast differentiation and migration of MSCs. Thus, the contents related to osteogenesis and migration might be the key to explaining the effect of ONFH-exos. We analyzed the proteome profiles of three random ONFH-exos and NOR-exos using Itraq-based LC-MS/MS proteomic technology. A total of 2702 proteins were identified, including 2636 in GO, 1420 in COG and 1786 in KEGG (Fig. 6a). Based on the differential expression thresholds and *P*-values < 0.05, 842 differentially expressed proteins (DEPs) were filtered out with 493 proteins upregulated and 349 proteins downregulated (Table S2). GO enrichment analysis of the 842 DEPs (Fig. 6b) showed that in molecular function analysis, a large part of the DEPs (49.63%) were associated with binding. In addition, we performed an analysis of the up- or downregulated pathway in KEGG to further study the biological behaviors of the DEPs (Fig. S4). DEPs in both upregulated and downregulated groups were mainly related to metabolic pathways.

To narrow the scope of DEPs, we focused on the 64 notable DEPs with more than eightfold-change (Table 1). The notable DEPs selected from the whole DEPs were showed in volcano plots (Fig. 6c) and these molecules were further analyzed employing cluster analysis (Fig. 6d). PRTN3, PERM, ITA2B (integrin α2b, CD41), et al. were reduced in ONFH-exos. Next, we reanalyzed the GO function of the 64 notable DEPs in ONFH-exos by circos map (Fig. 6e). From the notable proteins related to binding, we focused on integrins related to cell migration and osteogenic differentiation in MSCs^23,24^. ITA2B (CD41) also located at the core in interaction network performed by String analysis (Fig. S5). In brief, the proteomics data indicated that ITA2B (CD41) might play the pivotal role in the effects of ONFH-exos on MSCs.

**Table 1:** Notable DEPs in ONFH-exos with comparison of NOR-exos.

Accession to uniprot	Gene names	Protein names	Trend	Fold change	*P* value	Molecular function
P49747	COMP	Cartilage oligomeric matrix protein	Up	19.022	<0.01	Structural molecule activity; binding
P15088	CBPA3	Mast cell carboxypeptidase A	Up	18.953	<0.01	Binding; catalytic activity
P35442	TSP2	Thrombospondin-2	Up	17.779	<0.01	Binding
Q05707	COEA1	Collagen alpha-1(XIV) chain	Up	15.665	<0.01	Structural molecule activity; binding
P51884	LUM	Lumican	Up	15.468	<0.01	Structural molecule activity; binding
Q92954	PRG4	Proteoglycan 4	Up	14.757	<0.01	Molecular transducer activity; binding
P43652	AFAM	Afamin	Up	14.131	<0.01	Binding
P02774	VTDB	Vitamin D-binding protein	Up	13.849	<0.01	Transporter activity; binding
Q96PD5	PGRP2	N-acetylmuramoyl-L-alanine amidase	Up	11.946	<0.01	Molecular transducer activity; binding; catalytic activity
P55058	PLTP	Phospholipid transfer protein	Up	11.747	<0.01	Transporter activity; binding
P01859	IGHG2	Immunoglobulin heavy constant gamma 2	Up	11.123	<0.01	Binding
P51888	PRELP	Prolargin	Up	10.241	<0.01	Structural molecule activity; binding
Q14195	DPYL3	Dihydropyrimidinase-related protein 3	Up	10.109	<0.01	Binding; catalytic activity
Q15063	POSTN	Periostin	Up	10.017	<0.01	Binding
P35555	FBN1	Fibrillin-1	Up	9.926	<0.01	Structural molecule activity; binding
P28838	AMPL	Cytosol aminopeptidase	Up	9.897	<0.01	Binding; catalytic activity
Q06828	FMOD	Fibromodulin	Up	9.877	<0.01	Binding
P08603	CFAH	Complement factor H	Up	9.656	<0.01	Binding
P16112	PGCA	Aggrecan core protein	Up	9.41	<0.01	Binding
Q01995	TAGL	Transgelin	Up	9.15	<0.01	Binding
O43765	SGTA	Small glutamine-rich tetratricopeptide repeat-containing protein alpha	Up	9.132	<0.01	
P02545	LMNA	Prelamin-A/C	Up	9.078	<0.01	Structural molecule activity; binding
P02751	FINC	Fibronectin	Up	9.077	<0.01	Enzyme regulator activity; binding
P08582	TRFM	Melanotransferrin	Up	8.977	<0.01	Binding
Q15661	TRYB1	Tryptase alpha/beta-1	Up	8.97	<0.01	Binding; catalytic activity
P02790	HEMO	Hemopexin	Up	8.918	<0.01	Transporter activity; binding; catalytic activity
P00367	DHE3	Glutamate dehydrogenase 1, mitochondrial	Up	8.875	<0.01	Binding; catalytic activity
Q9NPH2	INO1	Inositol-3-phosphate synthase 1	Up	8.844	<0.01	Binding; catalytic activity
Q96CX2	KCD12	BTB/POZ domain-containing protein KCTD12	Up	8.838	<0.01	
P39059	COFA1	Collagen alpha-1(XV) chain	Up	8.836	<0.01	Structural molecule activity
P01009	A1AT	Alpha-1-antitrypsin	Up	8.707	<0.01	Enzyme regulator activity; binding
P00748	FA12	Coagulation factor XII	Up	8.656	<0.01	Binding; catalytic activity
P04216	THY1	Thy-1 membrane glycoprotein	Up	8.575	<0.01	Enzyme regulator activity; binding
P20774	MIME	Mimecan	Up	8.549	<0.01	Binding
Q9P015	RM15	39 S ribosomal protein L15, mitochondrial	Up	8.543	<0.01	Structural molecule activity; binding
P37802	TAGL2	Transgelin-2	Up	8.495	<0.01	Binding
Q92743	HTRA1	Serine protease HTRA1	Up	8.443	<0.01	Binding; catalytic activity
P10915	HPLN1	Hyaluronan and proteoglycan link protein 1	Up	8.407	<0.01	Binding
P12277	KCRB	Creatine kinase B-type	Up	8.259	<0.01	Binding; catalytic activity
P06396	GELS	Gelsolin	Up	8.134	<0.01	Binding
P05546	HEP2	Heparin cofactor 2	Up	8.125	<0.01	Enzyme regulator activity; binding
P00325	ADH1B	Alcohol dehydrogenase 1B	Down	0.112	<0.01	Binding; catalytic activity
P80188	NGAL	Neutrophil gelatinase-associated lipocalin	Down	0.112	<0.01	Transporter activity; binding
Q9HDC9	APMAP	Adipocyte plasma membrane-associated protein	Down	0.112	<0.01	Catalytic activity
Q9BRX8	F213A	Redox-regulatory protein FAM213A	Down	0.105	<0.01	Antioxidant activity
Q6PIU2	NCEH1	Neutral cholesterol ester hydrolase 1	Down	0.102	<0.01	Binding; catalytic activity
P00450	CERU	Ceruloplasmin	Down	0.1	<0.01	Binding; catalytic activity
P68366	TBA4A	Tubulin alpha-4A chain	Down	0.08	<0.01	Structural molecule activity; binding; catalytic activity
Q9HD89	RETN	Resistin	Down	0.079	<0.01	Binding
P28676	GRAN	Grancalcin	Down	0.077	<0.01	Binding; catalytic activity
P02786	TFR1	Transferrin receptor protein 1	Down	0.076	<0.01	Molecular transducer activity; transporter activity; binding
Q6UX06	OLFM4	Olfactomedin-4	Down	0.076	<0.01	Binding; catalytic activity
P08514	ITA2B	Integrin alpha-IIb	Down	0.075	<0.01	Binding
P12429	ANXA3	Annexin A3	Down	0.07	<0.01	Enzyme regulator activity; binding
P11678	PERE	Eosinophil peroxidase	Down	0.069	<0.01	Binding; catalytic activity; antioxidant activity
P15153	RAC2	Ras-related C3 botulinum toxin substrate 2	Down	0.067	<0.01	Enzyme regulator activity; binding; catalytic activity
P02788	TRFL	Lactotransferrin	Down	0.067	<0.01	Enzyme regulator activity; binding; catalytic activity
P06702	S10A9	Protein S100-A9	Down	0.054	<0.01	Molecular transducer activity; binding; antioxidant activity
P08311	CATG	Cathepsin G	Down	0.047	<0.01	Binding; catalytic activity
P05109	S10A8	Protein S100-A8	Down	0.044	<0.01	Binding; antioxidant activity
P59666	DEF3	Neutrophil defensin 3	Down	0.038	<0.01	
P20160	CAP7	Azurocidin	Down	0.037	<0.01	Binding; catalytic activity
P05164	PERM	Myeloperoxidase	Down	0.029	<0.01	Binding; catalytic activity; antioxidant activity
P24158	PRTN3	Myeloblastin	Down	0.018	<0.01	Binding; catalytic activity

Note: All the changes and the statistics analysis were displayed by ONFH-exos versus NOR-exos. *p* < 0.01 showed a statistical significance.

### ONFH-exos impaired osteoblast differentiation and migration of MSCs for lack of CD41, where CD41/integrin β3-FAK-Akt-Runx2 pathway was involved in

**Fig. 7:**
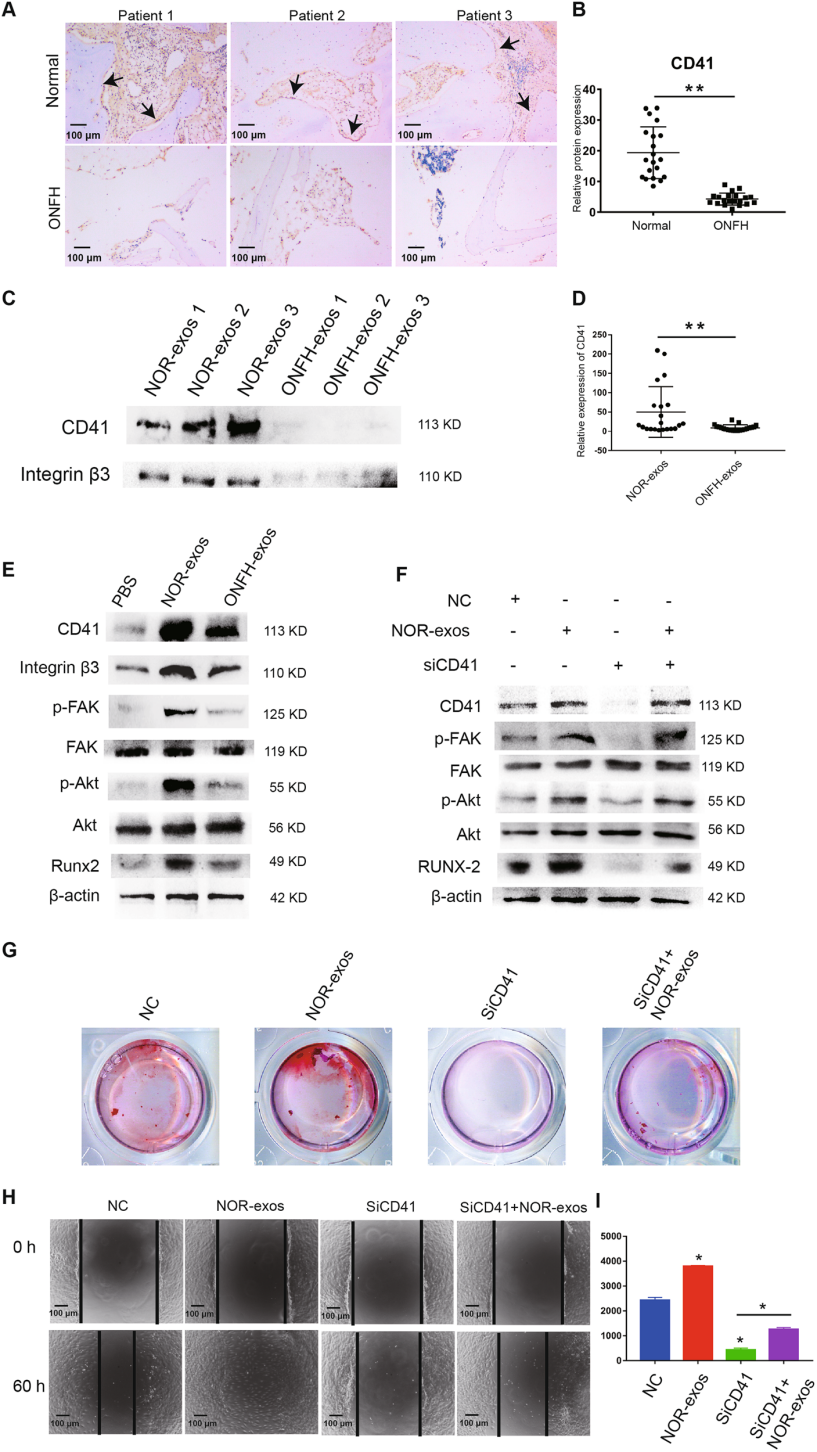
ONFH-exos impaired osteoblast differentiation and migration of MSCs for lack of CD41, where CD41/integrin β3-FAK-Akt-Runx2 pathway was involved in. **a** The decrease of CD41 in three human ONFH samples was confirmed by IHC, compared to the controls. **b** The quantitative results of CD41 in 20 ONFH samples and 20 control samples by IHC analysis further verified the decreased expression of CD41 in ONFH samples. **c** Declined expression of CD41 and integrin β3 between 3 ONFH-exos and 3 NOR-exos was validated by western blotting. **d** The quantitative results of CD41 in 21 ONFH-exos and 21 NOR-exos detected by western blotting. **e** C3H10T1/2 cells were pretreated with PBS, NOR-exos and ONFH-exos, for 48 h. The western blotting analysis showed protein levels of CD41, integrin β3, FAK, p-FAK, p-Akt, Akt, and Runx2 were downregulated in ONFH-exos group compared to NOR-exo group. **f** Western blotting analysis was performed to measure CD41, integrin β3, p-FAK, FAK, p-Akt, Akt, Runx2 in the control, NOR-exos, siCD41 and siCD41+ NOR-exos groups. **g** Calcium mineral deposits in C3H10T1/2 cells from the NC, NOR-exos, siCD41 and siCD41+ NOR-exos groups were measured by alizarin red S staining. **h** The migratory ability of C3H10T1/2 cells in NC, NOR-exos, siCD41 and siCD41+ NOR-exos groups was measured using wound healing assay, and the result showed that siCD41 led to the decline of MSCs migration and NOR-exos could rescue this decline. **i** Quantitative analysis of (**h**). **P < 0.01, versus normal specimens, *P < 0.05, versus normal specimens. All data were expressed as mean ± SEM.

To verify the data of proteomics, we first measured the protein level of CD41 in 20 human ONFH samples and control samples by IHC; the IHC images of three samples of each group were shown in Fig. 7a and the quantitative results of all the samples were displayed in Fig. 7b. The results exhibited that CD41 was declined in human ONFH samples. Then, we detected CD41 in 21 ONFH-exos and 21 NOR-exos by western blotting, and the results further suggested decrease of CD41 in ONFH-exos (Fig. 7c, d). The results of Fig. 7a–d demonstrated the declined expression of CD41 in ONFH bone tissue and ONFH-exos.

From the KEGG analysis result of DEPs, we found that CD41 and integrin β3 were correlated with focal adhesion pathway (Fig. S6). In this pathway, integrins activate the focal adhesion kinase (FAK) by phosphorylation, and then, p-FAK activates the phosphoinositide 3-kinase (PI3K)/protein kinase B (Akt). According to the previous study^25^, PI3K/Akt signaling fortifies the osteogenesis through increasing the expression of Runx2. Therefore, we assumed that ONFH-exos suppressed the osteogenic differentiation in MSCs via inactivating of CD41/integrin β3-FAK-PI3K/Akt-Runx2. We next detected CD41, FAK, p-FAK, Akt, p-Akt, and Runx2 in C3H10T1/2 cells after treated with PBS, NOR-exos, and ONFH-exos for 48 h (Fig. 7e). It was evident that ONFH-exos influenced the expression of these proteins compared with NOR-exos. These data suggested that ONFH-exos suppressed the osteogenic differentiation in MSCs via inactivating CD41/integrin β3-FAK-PI3K/Akt-Runx2 pathway.

Based on the results above, we supposed that ONFH-exos impaired CD41/integrin β3-FAK-PI3K/Akt-Runx2 pathway in MSCs for the lack of CD41. We performed a western blotting and alizarin red S staining to assess the effect of CD41 siRNA (siCD41) on osteogenic differentiation of MSCs (Fig. 7f, g). In NOR-exos group, the expression of CD41, p-FAK, p-Akt and Runx2 after 48-h treatment, and deposition of calcium after 14-day treatment were upregulated, while in siCD41 group, the results were reversed. In siCD41+NOR-exos group, after 48-h siCD41 treatment, C3H10T1/2 cells were incubated with NOR-exos for 48 h. The results showed that the decrease of CD41 and its downstream molecules as well as the deposition of calcium were restored by CD41-rich NOR-exos. To explore the importance of CD41 in MSCs migration, we observed the migration of C3H10T1/2 cells using a wound healing test after 48-h pretreatment (Fig. 7h, i). At 60 h, the migration of C3H10T1/2 cells were dramatically influenced by siCD41, but NOR-exos could repair the influence.

These data revealed that CD41/integrinβ3-FAK-PI3K/Akt-Runx2 pathway was important in osteoblast differentiation and migration of MSCs, and CD41 was a vital protein in activating this pathway. Additionally, NOR-exos with affluent CD41 might be an effective therapy to remedy the impaired osteogenic differentiation and migration of MSCs resulting from CD41 interference.

### Effects of NOR-exos on osteogenesis and migration of MSCs in vitro

**Fig. 8:**
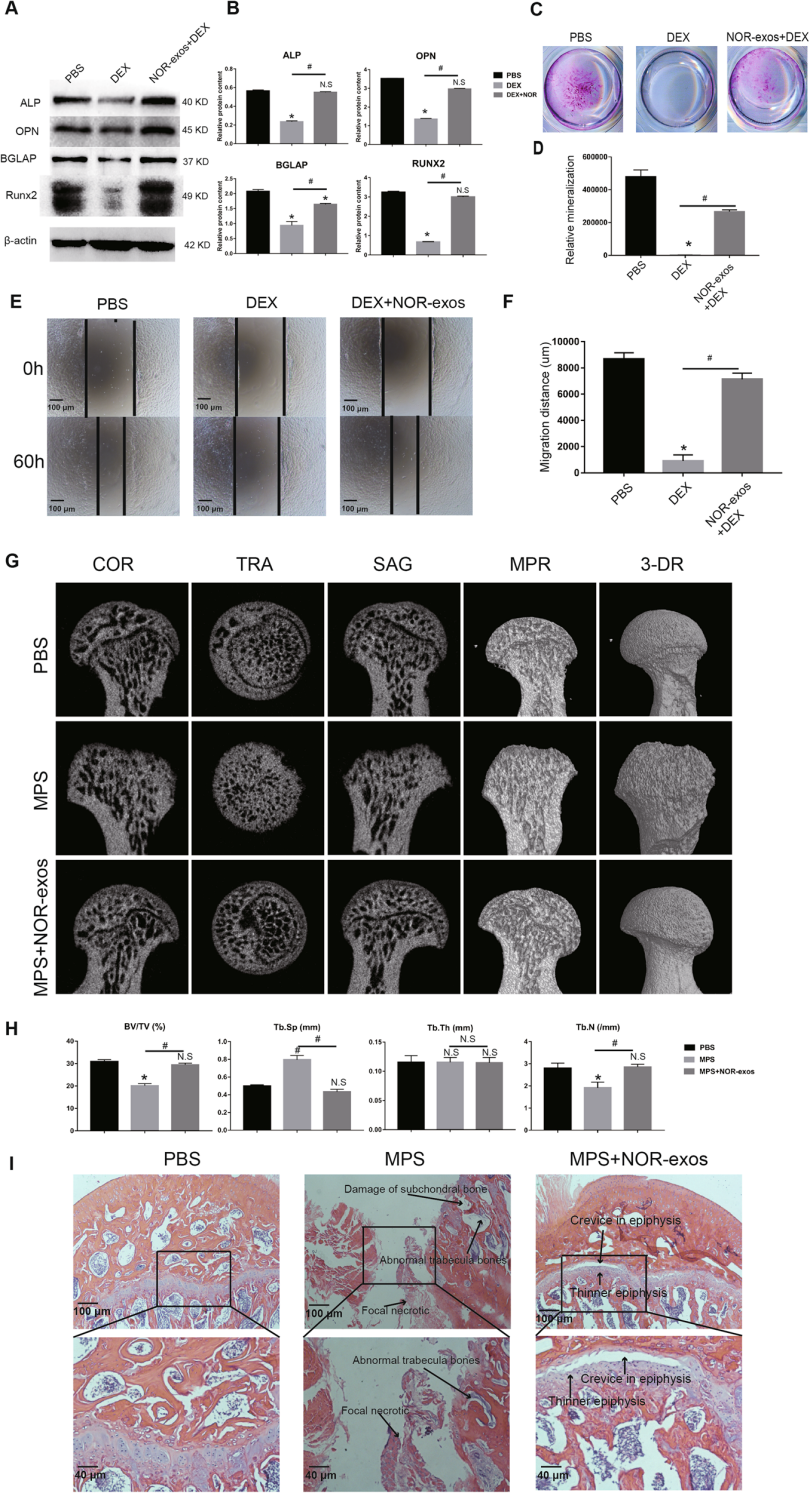
The therapeutic effect of NOR-exos on GC-induced ONFH. **a** The protein contents of ALP, OPN, BGLAP and Runx2 in PBS, DEX and NOR-exos+DEX group were assessed by western blotting. The results represented that DEX affected the expression of ALP, OPN, BGLAP and Runx2, but NOR-exos was able to improve the expression level of these proteins. **b** Quantitative analysis of (**a**). **c** Alizarin red S staining assay showed that NOR-exos were able to repair the damaged mineralization caused by DEX. **d** Displayed the quantity analysis of (**c**). **e**, **f** Wound healing assay was carried out to measure the migratory ability of C3H10T1/2 cells. The result showed that NOR-exos were able to restore the influenced migratory ability caused by DEX. **g** Reconstructed COR, TRA, SAG, MPR, and 3-DR images of femoral heads within the PBS, MPS, and NOR-exos+MPS groups were obtained using micro-CT scanning. In MPS group, the images of displayed damage of cartilage and subchondral bone and femoral head deformity. In NOR-exos+MPS group, although there was a small bone resorption zone in femoral head, no necrosis existed in femoral head. **h** Quantitative analysis of BV/TV, Tb.Sp, Tb.Th, and Tb.N were performed in the three groups. BV/TV, bone volume per tissue volume; Tb.N, trabecular number; Tb.Sp, trabecular separation; Tb.Th, Trabecular thickness. COR, coronal; TRA, transverse; SAG, sagittal; MPR, multiplanar reconstruction; 3-DR, three-dimensional reconstruction. **i** HE staining showed that MPS could cause ONFH in S-D rats, and NOR-exos could prevent ONFH in rats exposed to MPS. *P < 0.05, versus PBS group; ^#^*P* < 0.05, versus DEX group; N.S., no significance. All data were expressed as mean ± SEM.

The data of Fig. 7f showed that NOR-exos remedied the decrease of osteogenesis in MSCs caused by siCD41. So, we further evaluated the effect of NOR-exos in GC-induced ONFH-like damage model in vitro and in vivo (Fig. 8).

To assess the effects of the NOR-exos on osteogenesis of MSCs pretreated with DEX, the osteogenic differentiation ability of C3H10T1/2 cells was measured by western blotting (Fig. 8a). After 48-hour induction with DEX, the expression of ALP, OPN and Runx2 was dramatically decreased while after treatment of NOR-exos and DEX for 48 h, the results suggested that NOR-exos could restore the impaired osteogenesis in C3H10T1/2 cells caused by DEX (Fig. 8a, b). In addition, the alizarin red S staining demonstrated the similar effect of NOR-exos on calcium mineral deposits (Fig. 8c, d). The in vitro experiments confirmed the protective role of NOR-exos against DEX. Next, we evaluate the effect of NOR-exos on migration of DEX-treated C3H10T1/2 cells (Fig. 8e, f). As we expected, the NOR-exos could restore the impaired migration resulting from DEX. The data suggested that NOR-exos were able to restore the influence of DEX on osteogenesis and migration of C3H10T1/2 cells.

### Protective effects of NOR-exos on rats in vivo

The rats intravenously administrated with NOR-exos and intramuscularly injected with MPS simultaneously were used to observe the protective role of exosomes in vivo. Micro-CT scanning was conducted to evaluate the bone tissues within each of the rat femoral head (Fig. 8g). In MPS group, 60% rats showed significant trabecular changes, containing trabecula sparseness, subchondral area loss and malformation of the femoral heads. However, in NOR-exos+MPS group, no rats developed GC-induced ONFH-like damage, except one developed a small resorption focal in the left femoral head. Quantitative analysis of all the micro-CT parameters further validated the effect of the exosomes on osteogenesis (Fig. 8h). The results showed that BV/TV and Tb.N were markedly declined while Tb.Sp were notably accelerated in MPS group. NOR-exos could conspicuously reverse the reduction of BV/TV, Tb.N and augment of Tb.Sp caused by MPS. However, Tb.Th was constant in all the three groups.

Moreover, we detected the rats’ femoral heads by HE staining. The images in MPS group showed the damage of subchondral bone, abnormal structure of trabecula bones and homogeneous change in the focal necrotic zone. The images in MPS + NOR-exos group showed that the femoral head epiphysis was thinner and layered, but no focal necrosis in the femoral head (Fig. 8i). Overall, these data demonstrated that NOR-exos were able to treat GC-induced ONFH-like damage in vivo.

## Discussion

ONFH is a common disease that often causes disability in young vulnerable population, who are the major labor in society^26^. It is a heavy burden for the patients’ families and the society on financial^27^, because the early diagnosis is difficult and the conservative treatment is invalid. Currently, many studies of ONFH mechanism are focusing on the balance of osteogenesis, bone resorption and angiogenesis^28–31^. Among these mechanisms, the influenced osteogenic differentiation, proliferation and migration of MSCs are considered as the top factors leading to ONFH^8,9^. Glucocorticoid (GC)-treated models were used to study the mechanism of ONFH in vivo and in vitro, due to the use of GC as the common etiology of this disease^32^. However, cease of the GC administration fails to stop the exacerbation of ONFH clinically, which indicates that GC is an initial factor but not a maintaining factor Thus, the previous models can’t totally explain the progress of ONFH, because these studies failed to consider the influence of pathological microenvironment. Exosome, as an important carrier of signaling molecules in microenvironment, has attracted wide attention of researchers. The change of the contents might be related to some diseases, like osteoporosis^33^, and exosomes from MSCs were used to treat ONFH^34,35^. These studies suggested that exosomes were the crux in understanding the progress of disease. But the role of tissue-derived exosomes in ONFH is still unclear. In this study, we reported for the first time in the progress of ONFH, exosomes from the pathological bone brought about the failure of MSCs repairing the necrotic bone for lack of CD41, and prompted the progression of experimentally induced ONFH-like status in the rat. CD41 could be considered as the targets of early diagnosis and therapy in ONFH.

Exosomes exert diverse functions in bone remodeling via regulating osteogenic differentiation of MSCs and direct transfer of osteogenesis-related genes and proteins, according to Gao et al.^36^. As the bone microenvironment consist of multiple cell types, the tissue-derived exosomes are of multiple sources, mainly containing osteocytes, osteoblasts, MSCs, osteoclasts, adipocytes and vascular endothelial cells. Thus, the study of multi-source exosomes isolated from the local environment might be more proper for revealing the signal released from the pathological tissues. In this study, our results showed that ONFH-exos were able to induce GC-induced ONFH-like damage in S-D rats and impair the osteogenesis of MSCs. As the dysfunction of osteogenic differentiation of MSCs is a key process during ONFH^37^, it was inferred that ONFH-exos might cause ONFH by suppressing MSCs osteogenic differentiation. In addition, MSCs migrate to the damaged region to repair the bone damage^38^. The limited number of cells that migrate to the damaged region strongly restricts remedial effect of MSCs^39^. Our results showed that ONFH-exos could aggravate ONFH via affecting the migration of MSCs. In Fig. S3, ONFH-exos augmented the number of MSCs by increasing proliferation and decreasing apoptosis, which might be the compensation to the ischemic injury^40,41^. These data indicated that exosomes from the necrotic zone aggravated ONFH by influencing osteogenic differentiation and migration of MSCs.

Next, we analyzed the proteins in ONFH-exos using itraq-based proteomic technology and found that a large number of DEPs were enriched in binding function. Based on the GO enrichment of notable DEPs, the reported relation with osteogenesis and migration, we focused on the decrease of CD41. Furthermore, we showed that low expression of CD41 in ONFH-exos and testified the necessity of CD41 in MSCs osteogenic differentiation. Previous study showed that CD41 was required for osteoblastic differentiation caused by Ti microstructure and surface energy^42^. Upregulation of integrin β3, heterodimerized submit of CD41, could increase the osteoblastic differentiation^43^. Additionally, CD41, was reported to play an important role in migration of MSCs^23,44^. It is was suggested that the reduced level of CD41 in ONFH-exos might be the main cause for declined osteogenesis and migration of MSCs in ONFH. In our study, we also proved the accelerative effect of CD41-affluent NOR-exos in MSCs migration (Fig. 7h, i).

In our study, a total of 2702 proteins were identified in the tissue-derived exosomes, which is higher than the cell-derived exosomes^45^. There might be several causes: first, the sources of exosomes in tissues are more diverse than those in medium and serum; second, the number of non-exosomal protein contaminants measured by the proteomic system in these exosomes is higher; third, the itraq-base proteomic system in our study might be more sensitive than in previous study. But the larger number of exosome proteins might make it more difficult to find out the critical molecules and cell type in ONFH tissues than previous studies.

Our results in vitro and in vivo showed the therapeutic effects of NOR-exos on ONFH against GC and suggested that NOR-exos activated CD41/integrin β3-FAK-PI3K/Akt-Runx2 pathway to accelerate osteogenesis in MSCs, where the role of CD41 was critical. Luo et al. showed that the activation of the downstream FAK signaling pathway of integrin was coupled with robust MSC osteogenesis^46^. Akt is an important signal molecule in downstream of FAK. Previous studies have revealed that enhanced osteoinductivity was induced through activating the PI3K/Akt signaling pathway of human MSCs^47^, and this pathway regulated osteogenic differentiation^48^. Runx2 is the most essential transcription factor for bone formation^49^. These previous findings also supported that NOR-exos facilitated the osteogenesis in ONFH through activating CD41/integrin β3-FAK-Akt-Runx2 pathway. Also, it was inferred that ONFH-exos impaired the osteogenesis, due to inactivation of this pathway.

In conclusion, our results presented that exosomes from necrotic zone induced GC-induced ONFH-like damage by impairing the osteogenic differentiation for lack of CD41, experimentally induced ONFH-like status in the rat. The CD41-affluent nanoparticles can prevent the occurrence of ONFH-like damage in rats administered with GC via activating CD41/integrin β3-FAK-Akt-Runx2 pathway where CD41 played an important role. Our work indicated that the necrotic zone transmitted signals to the MSCs and damaged restorative capacity of MSCs. However, there were still some limitations in our work. First, some of the bone tissues were stored in the liquid nitrogen for a long time (within 1 year). Second, the exosomes derived from many different types of cells, thus, it is difficult to find the source of pathological signal. Third, high speed centrifugation may damage a part of exosomes^50^, although this isolation is considered as the gold standard and is the most commonly used techniques^51^. Last, the sample size of recruited patients in our study is small. In our future work, the role of ONFH-exos will be verified in more human ONFH tissues, and more in-depth mechanisms will be explored.

## Materials and methods

### Patients and bone tissues

This study was carried out in accordance with the Declaration of Helsinki. All experiments were approved by the Research Ethics Committee of the affiliated Hospital of Chongqing Medical University. Written informed consent was obtained from each donor with the permission of the Institutional Review Board of the First Affiliated Hospital of Chongqing Medical University.

The patients diagnosed with end ONFH or femoral neck fracture and decided to undergo a THA at the First Affiliated Hospital of Chongqing Medical University (Chongqing, China) were recruited from September 2017 to February 2019. For the recruited subjects, the inclusion criteria included were: (1) confirmed ONFH or fracture of the femoral neck diagnosis identified by plain radiographs, typical magnetic resonance imaging (MRI) and histological section; (2) ONFH group staged in the end period (stage III and IV) according to the ARCO Classification System^16^; (3) patients in ONFH group and control group willing to accept a THA; (4) no history of pharmacotherapy or surgery treatment in one year. The exclusion criteria for both groups included (1) concomitance with other bone diseases, such as metabolic bone diseases, Paget’s disease and osteoporosis; (2) patients who did not consent to this research program; (3) development of serious physical diseases prior to this project. All of these femoral head samples were collected after resection from the femur, and a part of the necrotic bone tissues and the counterpart of the normal bone tissues were rapidly stored in the liquid nitrogen until they were put into used. Meanwhile, another part of each biopsy specimen was obtained to analyze the histological performance and confirm the diagnosis. A total of 30 ONFH samples and 30 normal femoral head samples were collected in this study.

### Extraction of exosomes from ONFH and normal bone tissues

Based on the methods previously reported^52–55^, exosomes were extracted carefully from bone tissues after being homogenized by grinding. Briefly, 10-g aliquots of necrotic and normal bone tissues were disassociated into pieces, and homogenized in 20 mL phosphate buffer solution (PBS) supplemented with 200 μL complete Protease Inhibitor Cocktail (100×, Abcam) using a sterile mortar. The homogenate was transferred to centrifugal tube and centrifuged in series at low speeds (300 × *g* for 10 min, 2000 × *g* for 10 min, 10,000 × *g* for 30 min) to remove tissue and cell debris. After each centrifugation, the supernatant was collected for next step and the pellet was removed. Then, the final supernatant was filtered using a 0.2-μm pore filter (Millipore, USA) to discard large vesicles and the filtered liquid was collected. To further concentrate the exosomes, the filtered liquid was ultra-centrifuged at 100,000 × *g* for 70 min to pellet exosomes; then, the pelleted exosomes were washed thoroughly in 20 mL PBS and ultra-centrifuged again under the same condition for 70 min. After each ultracentrifugation, the supernatant was removed using pipette as much as possible. All steps above were carried out at 4 °C. Finally, the exosomes were dissolved in 100 μL sterile PBS and stored at −80 °C for the downstream experiments within 2 weeks. A part of exosome pellets were lysed in RIPA and PMSF lysis buffer (RIPA:PMSF = 100:1) and used for western blotting analysis.

### Characterization of exosomes

Samples were diluted 10,000-fold with filtered PBS. After that, the size distribution of exosomes was measured by nanoparticle tracking analysis (NTA) using Nanosizer^TM^ technology (Malvern). The data were processed with the Zeta View software. To observe the morphology, exosomes were loaded onto a 2-nm copper grid left to dry at room temperature for about 10 min and stained using phosphotungstic acid for 1 min. The grid was dried for 10 min at room temperature. The morphology of exosomes derived from both groups was visualized by a Hitachi H-7650 transmission electron microscope (TEM). The exosomal specific biomarkers including CD9, CD63, Flotillin-1, Alix and TSG101 were analyzed by western blotting. The expression of Lamin A and Mitofilin were analyzed by western blotting to further evaluate the purity of the exosomes. The ultra-centrifuged supernatant mentioned above was used as negative control (NC) in western blotting.

### Proteomics analysis of the ONFH and control exosomes

Three exosome samples from ONFH and three from control groups were randomly selected for proteomic screening. The procedures for sample preparation and iTRAQ-based proteomic analysis were reported in previous study^56^. Concisely, the samples were lysed by protein lysate and reduced by 10 mM dithiothreitol (DTT). After being ultrasounded and centrifuged, the supernate was precipitated by 100ul cold acetone overnight. The sediment was collected and redissolved re-dissolved by DTT for 30 min at 56 °C. Then, each sample was alkylated by iodoacetamide (IAM). The concentration of samples was measured by Bradford. A total of 100 μg of protein in every sample was digested using trypsin at room temperature overnight and the resulting peptides were desalted by C18 Empore (3 M) StAGE tips, and dried in vacuum. After that, the peptides were labeled according to iTRAQ8 kit (SCIEX) protocol. The three samples in control group were labeled with iTRAQ tag 115, 116, and 117. The three samples in ONFH group were labeled with iTRAQ tag 118, 119 and 120, respectively. The labeled samples were mixed and analyzed by Ultimate 3000 HPLC system (Thermo DINOEX, USA). Peptides were separated using the Durashell C18 column (5 μm, 100 Å, 4.6 × 250 mm) at a flow rate of 1 ml/min. Forty-two sub fractions were collected and merged into 12 components which were further desalted and dried on Strata-X column. The data were captured using a Triple TOF 5600 +LC-MS system (SCIEX). The peptides were dissolved in 0.1% formic loaded on the C18 column (5 μm, 100 μm × 20 mm) and analyzed with a Triple TOF 5600 plus mass spectrum combined with a Eksigent nanoLC system (SCIEX, USA). The peptides mixture were eluted on C18 analysis column with a 90 min gradient at a flow rate of 300 nL/min. The two mobile phases were composed by buffer A (0.1% formic acid and 2% acetonitrile in 98% distilled water) and buffer B (0.1% formic acid and 2% distilled water in 98% acetonitrile). The MS was scanned in ion accumulation time of 250 ms and the MS/MS of the 30 precursor ions were scanned in ion accumulation time of 50 ms. MS1 was recorded in a range of 350–1500 *m/z* and the MS2 was recorded in a range of 100–1500 *m/z* with an ion dynamic exclusion time of 15 s. The credible proteins were identified using the ABI Protein Pilot Software v4.5 with unused score ≥1.3 and inclusion of at least 1 unique peptides. The mass spectrometry proteomics data were deposited to the ProteomeXchange Consortium (http://proteomecentral.proteomexchange.org) via the iProX partner repository^57^ with the dataset identifier PXD018149.

The threshold value was set as >1.5 or <0.63 fold change and *p*-values < 0.05 in Student’s *t* test. The differential proteins were functionally annotated on the basis of their biological processes, cellular components and molecular functions by GO (gene ontology, http://www.geneontology.org) and COG (clusters of orthologous groups) annotation. Pathway analysis of these DEPs was performed with the KEGG online database (http://www.genome.jp/kegg/). Heat map of the significantly changed proteins was visualized using the R 2.11.1. Protein-Protein Interaction network was mapped using the online STRING 10.5 tool (https://string-db.org). Circos maps were created by circos 0.67.

### Cell culture and treatments

Murine MSC line C3H10T1/2 cells were purchased from the Cell Bank of the Chinese Academy of Sciences (Shanghai, China). All the cell lines were authenticated based on their cellular morphology and also by STR analysis, according to the guideline from ATCC. C3H10T1/2 cells were cultured in Dulbecco’s modified eagle medium (DMEM) suppled with 10% fetal bovine serum (FBS, Gibco), 100 U/mL penicillin and 100 μg/mL streptomycin (Gibco). The human primary bone marrow MSCs (HMSCs) were purchased from Zhong Qiao Xin Zhou Biotechnology (Shanghai, China). HMSCs were cultured in medium for human MSCs (Cyagen) containing 10% FBS and 1% dexamethasone.

To get an insight into the different effect of exosomes on MSCs osteogenesis, cells were treated with PBS, dexamethasone (DEX), NOR-exos, ONFH-exos. To explore the therapeutic effect of NOR-exos on ONFH, the HMSCs and C3H10T1/2 cells were treated with PBS, DEX, and DEX + NOR-exos. The concentration of DEX was 100 μM in apoptosis and 20 μM in other cell experiments. The concentration of NOR- and ONFH-exosomes added into cells was 50 μg/mL.

### Cell uptake of exosomes

The exosomes were stained by PKH67 kit (BestBio) according to the protocol. The labeled exosomes were dissolved in the sterile PBS. The C3H10T1/2 cells and HMSCs were treated by exosomes labeled by PKH67 and cultured in serum-free medium for 6 hours; then, the cells were washed by PBS and subsequently stabilized using paraformaldehyde for 10 min. Afterwards, the nuclei were stained by Diaminophenyl indole (DAPI) for 5 min and the redundant dye was washed off. At last, the cells were observed under a fluorescence microscopy (Leica DMI6000B).

### Effects of NOR-exos and ONFH-exos on osteoblastic bone formation

Osteogenesis of HMSCs and C3H10T1/2 cells were introduced by osteogenic differentiation medium (ODM) (Cyagen, Suzhou, China) based on the manufacturer’s instructions. HMSCs and C3H10T1/2 cells were seeded on culture plates (1.5 × 10^5^ cells/cm^2^) and cultured in ODM. Each group was prepared in triplicate. On Day 14, cells were photographed and confirmed by Alizarin Red S (0.2%, Cyagen) staining. The levels of osteogenic markers, involving alkaline phosphatase (ALP), osteocalcin (BGLAP), osteopontin (OPN) and collagen I (COL I), were assayed employing western blotting on Day 7, and RUNX2 was detected on Day 3.

### Effects of NOR-exos and ONFH-exos on adipogensis

C3H10T1/2 cells were seeded on 6-well plates (1.5 × 10^5^ cells/cm^2^) and cultured in adipogenic medium consisting of complete DMEM with 10 mg/ml insulin (Sigma-Aldrich), 25 mM 3-isobutyl-1-methylxanthine (Sigma-Aldrich), 10 mM dexamethasone (Sigma-Aldrich) and 6 Mm Indomethacin (Sigma-Aldrich). After 9-day treatment, cells were fixed with 4% paraformaldehyde for 30 min and rinsed twice with distilled water and once with 60% isopropanol, followed by staining with Oil Red O solution for 20 min. Adipocite differentiation of C3H10T1/2 cells were visualized under a light microscope, after washing with distilled water. The quantification of positive staining area demonstrating lipid droplets formation was performed using ImageJ software.

### Cells proliferation assay

To investigate the cells proliferation, CCK-8 (MedChem Express) assay and flow cytometry (FC) were used. HMSCs and C3H10T1/2 Cells (5 × 10^3^ cells per well) were seeded onto 96-well plates. A group without cells served as the blank. On the day 0, 1, 2, 4, and 6, 10 μL CCK-8 solution were added to HMSCs and incubated at 37 °C for 1 h; the blank group was processed in the same way. At 0, 12, 24, 36, and 48 h, CCK-8 solution was added to C3H10T1/2 cells and the blank group was put in the same condition. The absorbance was tested at 450 nm by a microplate reader and the real optical density (OD) values were the difference between the OD values of the cells in each well minus the OD value of the blank. For cell cycle analysis, HMSCs and C3H10T1/2 cells were serum-starved overnight and stimulated with PBS, DEX, NOR-exos, and ONFH-exos for 24 h. Cells were fixed in 75% ethanol overnight, stained with propidium iodide (PI, BD Biosciences, San Jose, CA) for 30 min and subsequently analyzed with FC. The cell cycle was analyzed by FlowJo software.

### Apoptosis assay

Apoptosis of HMSCs and C3H10T1/2 cells was researched in PBS, DEX, NOR-exos, ONFH-exos groups according to Annexin V-FITC/PI kit (GeneCopoeia) manual. The cells were seeded on 6-well plates with serum-free medium for 96 h, harvested and resuspended in 96 μL of Annexin V binding buffer, incubation followed by using Annexin V–FITC (1 μL) and PI (12.5 μL) for 10 min on ice in the dark. After that, the cell suspension was diluted with Annexin V binding buffer to a volume of 250 μL, and then measured by FC.

### Wound healing assay

Cell migration were detected by the wound healing experiment. The cells were plated on six-well plates (5 × 10^4^ cells/well) until 95–100% fusion, and the monolayer was scratched using a sterile pipette tip, and cultured with the serum-free medium. Wound width both at baseline, the images of cells in HMSCs at 24 h and in C3H10T1/2 at 60 h were obtained via optical microscopy (Olympus Corporation, Tokyo, Japan) to determine the migration ability of cells in each group.

### Western blotting

Protein samples were harvested and their concentrations were analyzed by BCA protein assay kit (Bi Yun tian, China). In brief, total protein exacts were separated on a sodium dodecyl sulfate-polyacrylamide gel electrophoresis (SDS-PAGE) gel, in Tris Buffered Saline Tween (TBST). After electrophoresis, the separated proteins were transferred to a polyvinylidene fluoride (PVDF) membranes (Merk Millipore) at 210 mA for 1.5 h and then blocked with 5% blocking protein ponder (Boster Biology Technology) for 1 h at room temperature. Next, the PVDF membrane was incubated in the primary antibody overnight at 4 °C. Next, the membranes were washed in TBST, and incubated in secondary antibodies for 1 h.

Primary antibodies used in our study, involving Akt (4691), phospho-Akt (Ser473) (9271) and integrin β3 (13166), were purchased from Cell Signaling Technology; ALP (108337), FAK (40794), phosphor-FAK (Y397) (81298), CD41 (134131), Runx2 (92336), CD9 (92726), Flotillin-1 (133497), TSG101 (125011), Mitofilin(110329), Lamin A (8984) and GAPDH (181602) were obtained from Abcam; OPN (02948), CD63 (02549), collagen I (0088) and Alix (03338) were purchased from WanLei Biotechnology; β-actin (30345) was obtained from ComWin Biotechnology and BGLAP (60987) from Bioworld Technology. Protein quantification was performed by Image J.

### RNA interference

To probe into the role of CD41, small interfering RNA (siRNA, GenePharma, Shanghai) was used to knock down intracellular CD41. C3H10T1/2 cells were seeded onto six-well plate and cultured in serum-free ODM for 24 h. Entranster^TM^-R4000 (Engreen Biosystem, Beijing) was used to transfer CD41 siRNA and NC according operation manual. After 48 h, the cells were collected for next western blotting measurement. To assess the role of CD41 in MSCs migration, C3H10T1/2 cells were seeded onto six-well plate and cultured in serum-free DMEM for 60 h, the wound healing assay was performed.

### In vivo effects of ONFH-exos and NOR-exos on rats

All experimental and animal care procedures were approved by Animal Research Ethics Committee of The First Affiliated Hospital of Chongqing Medical University, and carried out in accordance with the guidelines of the National Institutes of Health Guidelines for the Care and Use of Laboratory Animals.

All the rats were purchased form the Animal Laboratory in Chongqing Medical University, and the animal experiments were performed in accordance with the previous study^35^. Briefly, to evaluate the effects of ONFH-exos, thirty 6-week-old healthy female Sprague-Dawley (S-D) rats weighing from 140 to 160 g were equally and randomly divided into PBS group, NOR-exos group and ONFH-exos group. Furthermore, another 30 S-D female rats were equally and randomly divided into PBS group, methylprednisolone (MPS) group and NOR-exos+MPS group. In MPS group, MPS (20 mg/kg/d) was injected intramuscularly into rats on the first three days of every week for three weeks. Rats in PBS group were injected with 200 μL PBS through tail veins. The NOR-exos group and ONFH-exos group were respectively administered with 100 μg (dissolved in 200 μL PBS) NOR-exos and ONFH-exos. In NOR-exos+MPS group, each time after MPS injection, 100 μg NOR-exos were used to restore the influence of MPS. Two months after treatment, the rats were sacrificed, and then the femoral heads were isolated for micro-CT examination and HE-staining. The assessors in micro-CT scan and HE staining of rats were blinded to the group allocation during the experiment and/or when assessing the outcome.

Micro-CT (Skyscan1174 X-Ray Microtomograph, Bruker, Belgium) were used to scan the rat femoral heads. After scanning, software N-Recon were used for 3-demetional reconstruction of the femoral heads and software CT-AN were used to analyze the osteogenic parameters including BV/TV (bone volume per tissue volume), Tb.Sp (trabecular separation), Tb.Th (trabecular thickness) and Tb.N (trabecular number).

### Histological analyses and immunohistochemistry (IHC)

The data were expressed as means ± standard deviation (SD). The difference of proliferation rates was determined by one-way analysis of variance (ANOVA). Mann–Whitney *U*-test, Wilcoxon matched-pairs test and Student’s *t*-test were conducted to compare means between two different groups. Statistical analysis was performed using GraphPad Prism 7.0 and *P* values < 0.05 were considered statistically significant. Semiquantitation of western blotting, IHC, wound healing and alizarin red S staining, was performed by Image J.

### Statistical analysis

The data were expressed as means ± standard deviation (SD). The difference of proliferation rates was determined by one-way analysis of variance (ANOVA). Mann–Whitney *U*-test, Wilcoxon matched-pairs test and Student’s t-test were conducted to compare means between two different groups. Statistical analysis was performed using GraphPad Prism 7.0 and *P* values < 0.05 were considered statistically significant. Semiquantitation of western blotting, IHC, wound healing and alizarin red S staining, was performed by Image J. All the experiments consisted of at least three replicates.

## Supplementary information

Supplementary Material Legends

Table S1

Table S2

Figure S1

Figure S3

Figure S2

Figure S4

Figure S5

Figure S6
