# Supplementary material for: CD41-deficient exosomes from non-traumatic femoral head necrosis tissues impair osteogenic differentiation and migration of mesenchymal stem cells
Source: Cell Death Dis. 2020 Apr 27;11(4):293. doi: 10.1038/s41419-020-2496-y (PMC7184624; doi:10.1038/s41419-020-2496-y)
Supplement: Supplementary file 1 — Supplementary Material Legends [file 41419_2020_2496_MOESM1_ESM.docx]

**CD41-deficient exosomes from non-traumatic femoral head necrosis tissues impair osteogenic differentiation and migration of mesenchymal stem cells**

Weiwen Zhu^a^, MinKang Guo^a^, Wu Yang ^a^, Min Tang^b^, Tingmei Chen^b^, Delu Gan^b^, Dian Zhang^b^, Xiaojuan Ding^c^, Anping Zhao^a^, Pei Zhao^a^, Wenlong Yan^a^, Jian Zhang^a^*

a. Department of Orthopedics, The First Affiliated Hospital of Chongqing Medical University, Chongqing 400016, China.

b. Key Laboratory of Diagnostic Medicine Designated by the Ministry of Education, Chongqing Medical University, Chongqing 400016, China

c. Clinical Laboratory, The Second Affiliated Hospital of Chongqing Medical University, Chongqing 400016, China.

* Corresponding author at: Department of Orthopedics, the First Affiliated Hospital of Chongqing Medical University. 1st Youyi Road, Yuzhong, Chongqing 400016, China.

E-mail address: zhangjian@hospital.cqmu.edu.cn (Jian Zhang)

**Table S1. Demographics data of the study groups.** Data are presented as mean ± standard deviation (SD). “ONFH” represents the group of osteonecrosis of the femoral head. “BMI” means body mass index.

**Table S2. Proteomics data of the 842 DEPs in ONFH-exos.** P value was obtained using Student’s t test. “Unused” represents the confidence level of the identified protein. “%Cov(95)” represents Protein sequence coverage (95% confidence). “#of Unique Peptides” represents the number of unique peptides contained in the protein. “#of Peptides” represents number of all the peptides contained in a protein. “#of Spectra” represents number of spectra contained in a protein. “#of Unique Spectra” represents number of unique spectra contained in a protein.

**Figure S1. Lamin A and mitofilin were analysed in NOR-exos and ONFH-exos using western blotting.** The results showed the low expression of lamin A and mitofilin in NOR-exos and ONFH-exos. “NOR-HOM”: the normal bone tissue generate; “ONFH-HOM”: the ONFH bone tissue generate.

**Figure S2. Effect of ONFH-exos on adipogenesis of C3H10T1/2 cells cultured in adipogenic medium**. **a** The adipogenesis of C3H10T1/2 cells were measured using oil red O staining in PBS group, NOR-exos group and ONFH-exos group. The results presented an obvious increase in ONFH-exos group. **b** The quantitation results of lipid droplets in **a.** *P<0.05, versus PBS group, all data were expressed as mean ± SEM.

**Figure S3**. **Effect of NOR-exos and ONFH-exos on proliferation and apoptosis in C3H10T1/2 cells and HMSCs**. **a** The CCK-8 test displayed the increment of cell viability in ONFH-exos group compared to NOR-exos group. **b** The cell circle analysis and of C3H10T1/2 cells and HMSCs assessed by flow cytometery (FC) showed the increase of cell number in phase S in ONFH-exos group. **c** The percentage of cell number in phase S, based on the result above. *P<0.05, versus PBS group; N.S, no significance versus PBS group. All data were expressed as mean ± SEM. **d** Apoptosis of C3H10 cells and HMSCs induced by DEX, NOR-exos and ONFH-exos assessed through Annexin V-FITC/PI double staining with flow cytometric analysis. *P<0.05, versus PBS group, all data were expressed as mean ± SEM.

**Figure S4. GO analysis of up-regulated and down-regulated proteins**. In the up-regulated and down-regulated proteins, the set of metabolic pathways is the most important one in all GO sets.

**Figure S5. String analysis of the notable DEPs**. Protein–protein interaction networks of differentially expressed proteins in ONFH-exos using the web-based tool STRING v11.0 (<https://string-db.org/>).

**Figure S6.** **The down-regulated DEPs in focal adhesion pathway analyzed by KEGG (**[**http://www.genome.jp/kegg/**](http://www.genome.jp/kegg/)**).**
