# Supplementary material for: CD41-deficient exosomes from non-traumatic femoral head necrosis tissues impair osteogenic differentiation and migration of mesenchymal stem cells
Source: Cell Death Dis. 2020 Apr 27;11(4):293. doi: 10.1038/s41419-020-2496-y (PMC7184624; doi:10.1038/s41419-020-2496-y)
Supplement: Supplementary file 2 — Table S1 [file 41419_2020_2496_MOESM2_ESM.docx]

**Table S1 Demographics data of the study groups**

| **group** | **gender (male/female)** | **side (right/left)** | **age**  **(years)** | **BMI**  **(kg/m^2^)** | **Acro stage** | **Yield of exosomes (10^11^/g)** |
| --- | --- | --- | --- | --- | --- | --- |
| ONFH | 19/11 | 16/14 | 64.3±8.1 | 22.15±1.7 | stage Ⅲb (n=4)  stage Ⅲc (n=6)  stage Ⅳ (n=20) | 5.38±1.75 |
|  |  |  |  |  |  |  |
|  |  |  |  |  |  |  |
| Control | 18/12 | 14/16 | 65.7±5.8 | 22.18±1.8 |  | 2.8±0.85 |

Note: Data are presented as mean ± standard deviation (SD). “ONFH” represents the group of osteonecrosis of the femoral head. “BMI” means body mass index.
