# Supplementary figures and images for: CD41-deficient exosomes from non-traumatic femoral head necrosis tissues impair osteogenic differentiation and migration of mesenchymal stem cells
Source: Cell Death Dis. 2020 Apr 27;11(4):293. doi: 10.1038/s41419-020-2496-y (PMC7184624; doi:10.1038/s41419-020-2496-y)

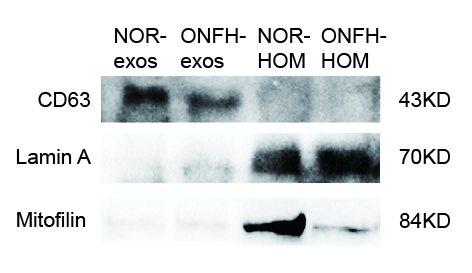

Supplement: Supplementary file 4 — Figure S1 [file 41419_2020_2496_MOESM4_ESM.tif]

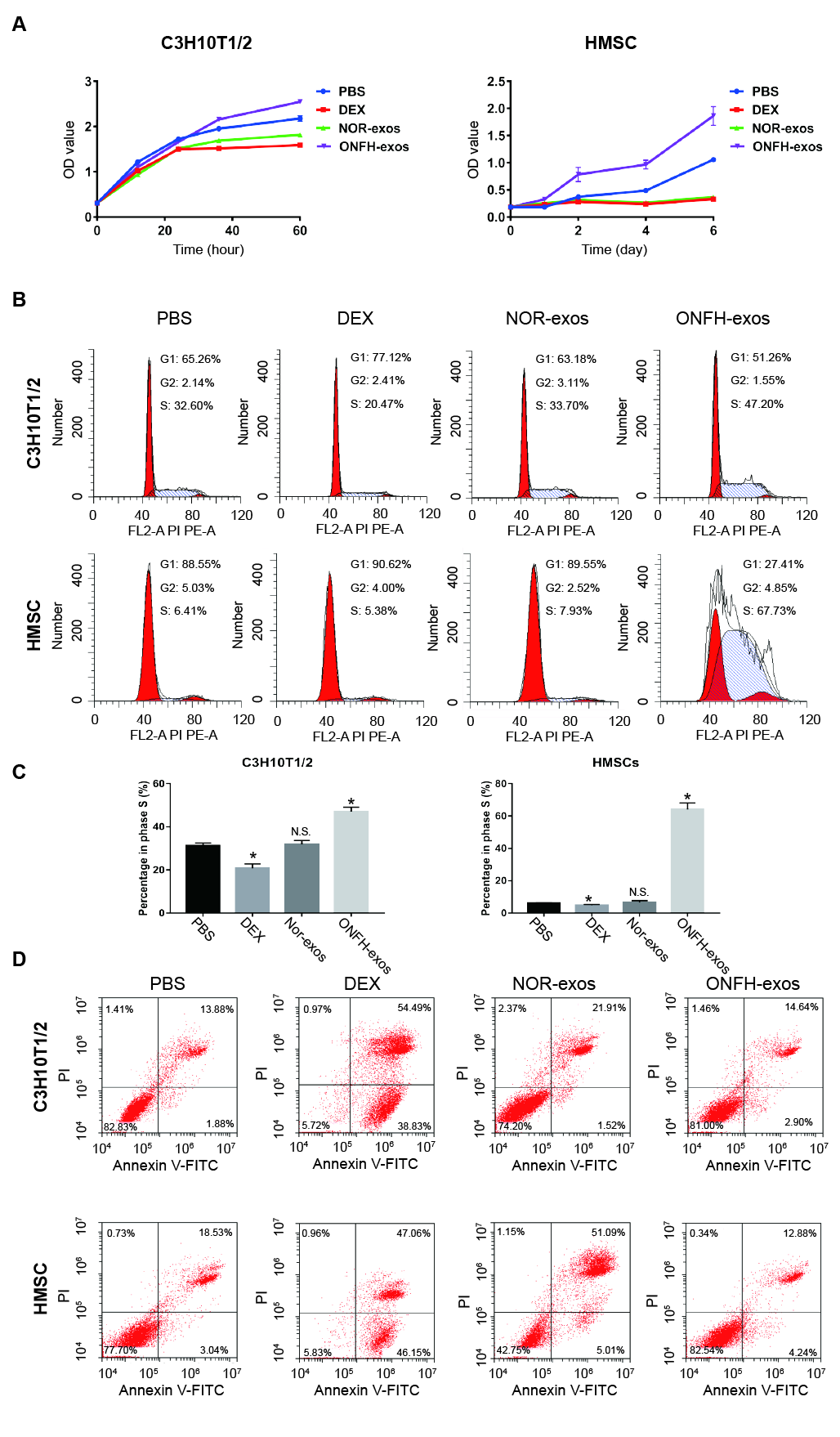

Supplement: Supplementary file 5 — Figure S3 [file 41419_2020_2496_MOESM5_ESM.tif]

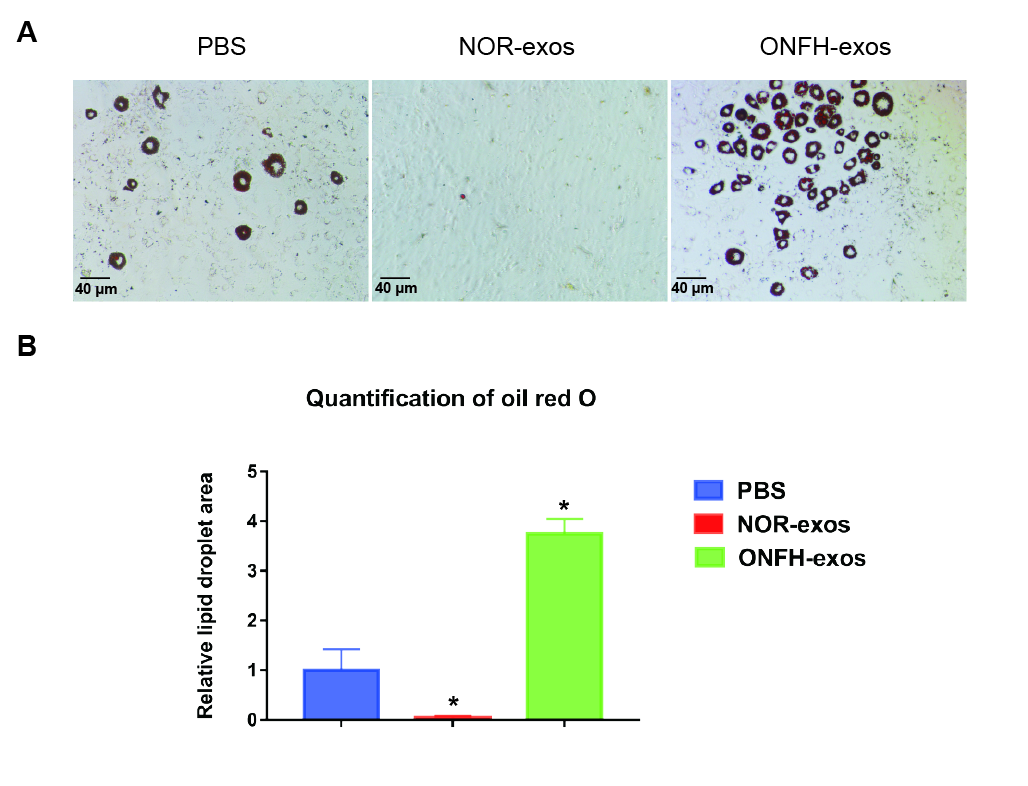

Supplement: Supplementary file 6 — Figure S2 [file 41419_2020_2496_MOESM6_ESM.tif]

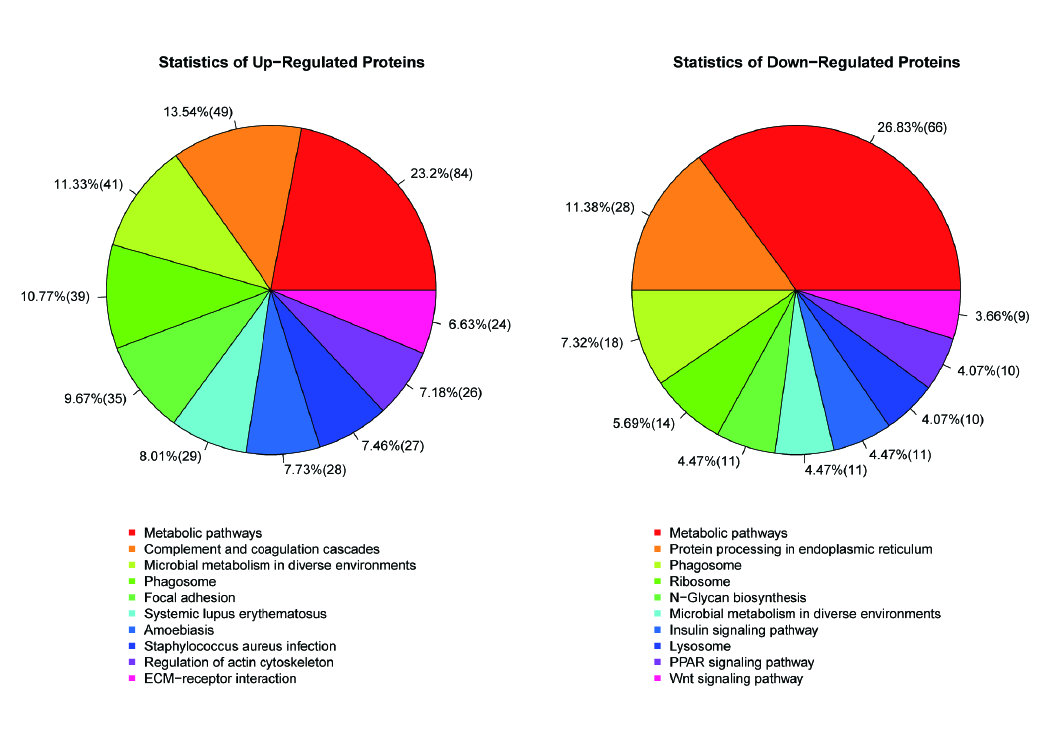

Supplement: Supplementary file 7 — Figure S4 [file 41419_2020_2496_MOESM7_ESM.tif]

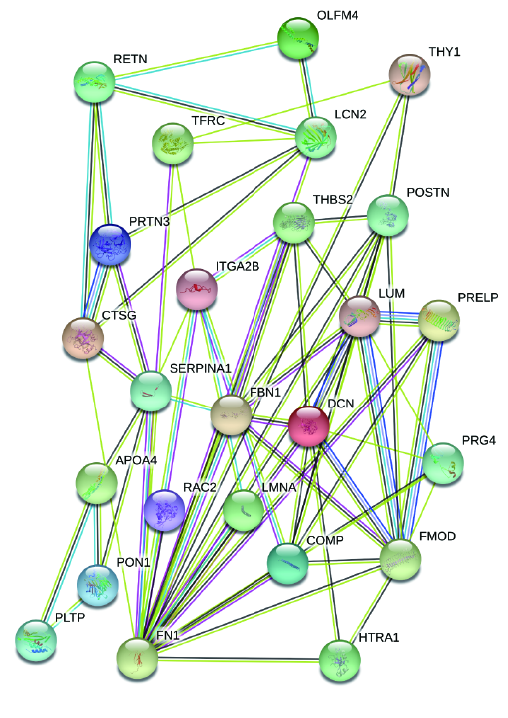

Supplement: Supplementary file 8 — Figure S5 [file 41419_2020_2496_MOESM8_ESM.tif]

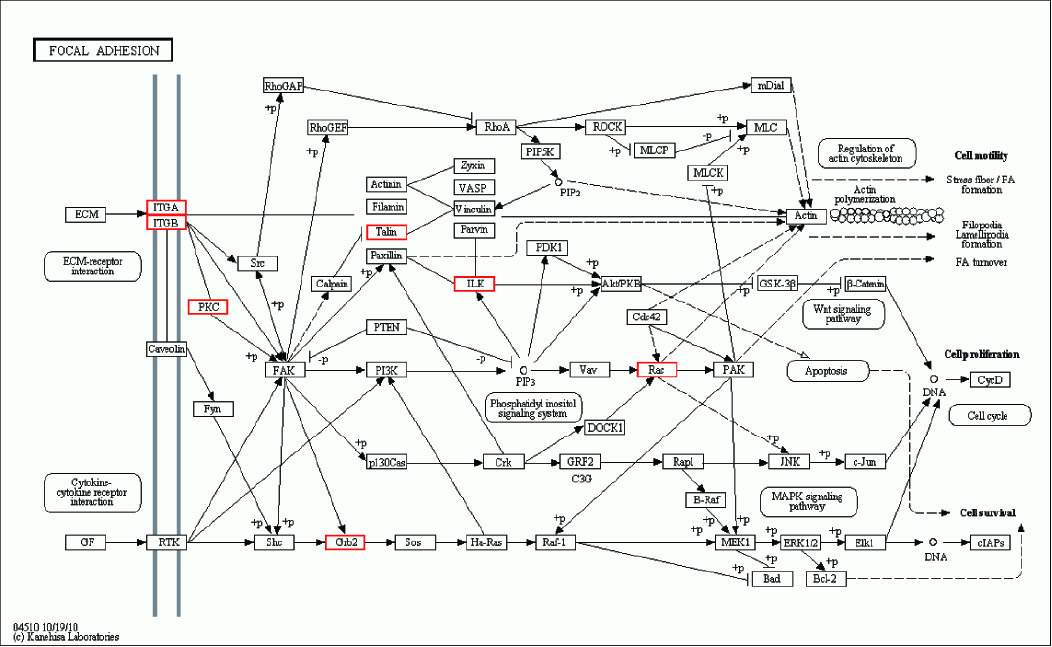

Supplement: Supplementary file 9 — Figure S6 [file 41419_2020_2496_MOESM9_ESM.tif]
